# A Chemical Investigation of the Leaves of *Morus alba* L.

**DOI:** 10.3390/molecules23051018

**Published:** 2018-04-26

**Authors:** Xiao-yan Chen, Ting Zhang, Xin Wang, Mark T. Hamann, Jie Kang, De-quan Yu, Ruo-yun Chen

**Affiliations:** 1 Department of Drug Discovery and Biomedical Sciences, College of Pharmacy, Medical University of South Carolina, Charleston, SC 29425, USA; chenxiaoyan8615@gmail.com or chenxi@musc.edu (X.-y.C.); hamannm@musc.edu (M.T.H.); 2 Institute of Medical Information & Library, Chinese Academy of Medical Sciences and Peking Union Medical College, Beijing 100020, China; brendatingting@126.com; 3 Beijing Key Laboratory of Bioactive Substances and Function Foods, Beijing Union University, Beijing 100191, China; shtwangxin@buu.edu.cn; 4 State Key Laboratory of Bioactive Substance and Function of Natural Medicines, Institute of Materia Medica, Chinese Academy of Medical Sciences and Peking Union Medical College, Beijing 100050, China; jiekang@imm.ac.cn (J.K.); dqyu@imm.ac.cn (D.-q.Y.)

**Keywords:** *Morus alba* L., aldose reductase inhibitor, neuroprotective agent, natural products

## Abstract

The leaves of *Morus alba* L. are an important herbal medicine in Asia. The systematic isolation of the metabolites of the leaves of *Morus alba* L. was achieved using a combination of liquid chromatography techniques. The structures were elucidated by spectroscopic data analysis and the absolute configuration was determined based on electronic circular dichroism (ECD) spectroscopic data and hydrolysis experiments. Their biological activity was evaluated using different biological assays, such as the assessment of their capacity to inhibit the aldose reductase enzyme; the determination of their cytotoxic activity and the evaluation of their neuroprotective effects against the deprivation of serum or against the presence of nicouline. Chemical investigation of the leaves of *Morus alba* L. resulted in four new structures **1**–**4** and a known molecule **5**. Compounds **2** and **5** inhibited aldose reductase with IC_50_ values of 4.33 μM and 6.0 μM compared with the potent AR inhibitor epalrestat (IC_50_ 1.88 × 10^−3^ μM). Pretreatment with compound **3** decreased PC12 cell apoptosis subsequent serum deprivation condition and pretreatment with compound **5** decreased nicouline-induced PC12 cell apoptosis as compared with control cells (*p* < 0.001).

## 1. Introduction

The species *Morus alba* L., known as white mulberry, belongs to the genus *Morus* of the family Moraceae, is native to China and now is cultivated throughout the world [1,2]. All parts of this plant have been used medicinally in Traditional Chinese Medicine including the leaves, root bark, stem and fruits [3,4]. In East Asia the leaves have been an important herbal medicine for treatment of cold, fever, headache, cough and rheumatic diseases for thousands of years [3,5]. Extracts or constituents of *M. alba* L. leaves were reported to possess anti-inflammatory, antioxidant, antiobesity, antidiabetic, and hypolipidemic properties [5,6]. Phytochemical investigations of the leaves of *M. alba* reported the presence of flavonoids, lignans, pyrrole alkaloids, polyphenols, fatty acids, and anthocyanin [6,7]. Previous phytochemical studies and the pharmacological potentials of constituents of the genus *Morus* have been reviewed by Yang et al. [8].

Aldose reductase is the first and rate-controlling enzyme in the polyol pathway that reduces glucose into sorbitol and then fructose. Intracellular excess sorbitol is thought to lead to diabetic complications, including neuropathy, nephropathy, retinopathy, and cataract [9]. Fructose can lead to the formation of 3-deoxyglucosone, a key intermediate known to accelerate the formation of Advanced Glycation End products (AGEs) [10]. The presence and accumulation of AGEs contribute to the development of atherosclerosis and promotes renal damage, diabetic nephropathy, and a series of cancers [11,12,13,14]. Besides reducing glucose, aldose reductase is also involved in the reduction of oxidative stress-generated lipid aldehydes and their conjugate with GSH, which can alter cellular signals by mediating transcription factors such as NF-Kb and AP1 [15]. Aldose reductase inhibitors have been shown to be an effective multi-disease target to prevent diabetic complications, cancers, cardiovascular diseases, and inflammatory complications [16]. This study investigated the acetone and chloroform fractions of the ethanol extract of the leaves of *M. alba* L., which showed effects against diabetes and human cancer cell lines in our previous study, leading to the identification of four new structures, namely a sesquiterpenoid glucoside **1**, an aromatic glucoside **2**, a farnesylacetone derivative **3**, a flavan **4**, and a known compound, (9*R*)-hydroxyl-(10*E*,12*Z*,15*Z*)-octadecatrienoic acid (**5**). In addition, we report the results of aldose reductase inhibitory and neuroprotective activity evaluations. 

## 2. Results and Discussion

### 2.1. Characterization

**Figure 1:**
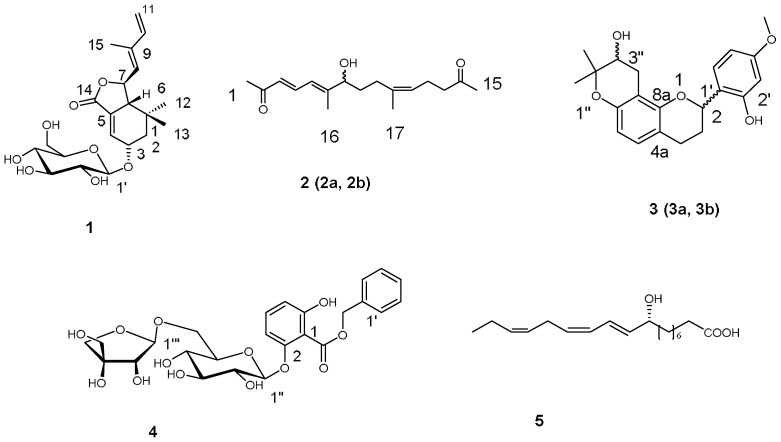
Structures of Compounds **1**–**5**.

The crude extract of the leaves of *Morus alba*. L was divided into four fractions by flash silica gel column chromatography. The generated acetone and chloroform fractions were further isolated by the combination of resin column chromatography, silica gel column chromatography, medium pressure liquid chromatography (MPLC), and high performance liquid chromatography (HPLC), generating four new compounds and a known one (Figure 1). 

Moralsin (**1**) was obtained as a white powder, ${\left[\alpha \right]}_{D}^{20}$ −72 (*c* 0.19, MeOH). The IR spectrum of **1** showed the presence of hydroxy (2957 cm^−1^), alkyl (3402 cm^−1^) and ester carbonyl (1760 cm^−1^) functional groups. The molecular formula, C_21_H_30_O_8_, was determined from its sodium adduct ion in the HRESIMS (433.1826 [M + Na]^+^, calcd. 433.1833), corresponding to seven indices of hydrogen deficiency. The ^1^H-NMR and ^13^C-NMR spectra (Table 1 and Table 2) revealed the presence of two trisubstituted double bonds [δ_H_ 6.82 (t, *J* = 3.0 Hz, H-4), 5.66 (d, *J* = 9.3 Hz, H-8); δ_C_ 132.4 (C-4), 130.7 (C-5), 129.8 (C-8), 138.8 (C-9)], one terminal double bond [δ_H_ 6.44 (dd, *J* = 17.5, 10.5 Hz, H-10), 5.37 (d, *J* = 17.5 Hz, H-11a), 5.20 (d, *J* = 10.5 Hz, H-11b)], one ester carbonyl (δ_C_ 168.8), two oxygenated methines [δ_H_ 4.41 (m, H-3), 5.12 (t, *J* = 9.3 Hz, H-7); δ_C_ 70.4 (C-3), 76.8 (C-7)], three methyls [δ_H_ 0.90 (3H, s), 0.87 (3H, s), 1.88 (3H, s)], one methylene [δ_H_ 1.86 (m, H-2α), 1.60 (dd, *J* = 15.0, 6.5 Hz, H-2β)], two methines [δ_H_ 2.57 (m, H-6), 5.12 (t, *J* = 9.3 Hz, H-7)], one tertiary carbon group [δ_C_ 29.5 (C-1)] and a glucopyranosyl unit [δ_H_ 4.35 (d, *J* = 8.0 Hz, H-1′), 2.9-3.7 (6H, H-2′-6′)]. In combination with analysis of ^1^H-^1^H COSY spectrum, the NMR date displayed there were two spin systems C2-C3-C4 and C6-C7-C8 in **1**. In the HMBC spectrum, the correlations of H-12, H-13/C-1, C-2, C-6; H-4/C-5, C-14; H-6/C-4; H-11/C-9; and H-15/C-8, C-10 determined the monocyclofarnesane carbon skeleton containing a 14,7-olide ring. In addition, the correlations of H-1′/C-3 indicated the glucopyranosyl unit was connected to C-3 of the monocyclofarnesane-type sesquiterpenoid aglycone.

**Figure 2:**
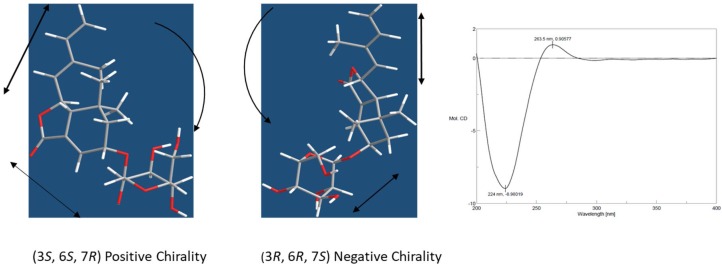
ECD Spectrum of compound **1.** The positive Cotton effect of ECD spectrum is in agreement with the negative chirality of (3*S*, 6*S*, 7*R*) diastereoisomer of compound **1**.

A β-anomeric configuration for the glucosyl unit was assigned via its large ^3^*J*_1,2_ coupling constant (8.0 Hz). The D-configuration of the glucose was determined by GC analysis of the trimethylsilyl l-cysteine derivatives after acid hydrolysis of **1**. The *E*-configuration of the 8,9-double bond was demonstrated by the nuclear Overhauser effect (NOE) effect of H-7/H-15 and H-8/H-10 in the ROESY 1D experiment. NOE effect of H-3/H-2β, H-7/H-12, and H-6/H-8, H-2β, and H-13 showed that H-2β, H-3, H-6, C-13, and C-8 were cofacial, assigned as the β-orientation, and H-7 and C-12 were α-oriented. The absolute configuration of aglycone moiety was assigned by analysis of the electronic circular dichroism (ECD) spectroscopy using excitation chirality method [17]. The ECD spectrum showed positive Cotton effect at 263 nm and negative Cotton effect at 224 nm arising from coupling between conjugated diene and α,β-unsaturated ester chromophores (Supplementary Materials). Such a pattern was in agreement of a negative chirality of **1** as depicted in Figure 2. Thus the absolute configuration of **1** was unequivocally assigned as (3*S*, 6*S*, 7*R*) and the structure of moralsin was determined as **1**.

Compound **2** was obtained as a yellow oil. The IR spectrum of **2** showed the presence of hydroxy (3419 cm^−1^), alkyl (2931 cm^−1^), and carbonyl (1712 cm^−1^) functional groups. The molecular formula, C_17_H_26_O_3_, was determined from its sodium adduct ion in the HRESIMS (301.1785 [M + Na]^+^, calcd. 301.1774), corresponding to five indices of hydrogen deficiency. The ^1^H-NMR (Table 1), ^13^C-NMR (Table 2), and DEPT (Supplementary Information) spectra revealed the presence of three double bonds at δ_H_ 6.09 (d, *J* = 16.5 Hz, H-3), 7.45 (dd, *J* = 16.5, 11.5 Hz, H-4), 6.23 (d, *J* = 11.5 Hz, H-5), 5.03 (t, *J* = 7.0 Hz, H-11) and δ_C_ 129.9 (C-3), 139.4 (C-4), 122.3 (C-5), 153.1 (C-6), 135.3 (C-10), 124.0 (C-11), a oxymethine group at δ_H_ 3.93 (t, *J* = 6.5 Hz, H-7), four methylene groups at δ_H_ 1.47 (m, H-8), 1.97 (t, *J* = 7.5 Hz, H-9), 2.12 (m, H-12), 2.42 (t, *J* = 7.5 Hz, H-13), four terminal methyl groups at δ_H_ 2.25 (s, H-1), 2.06 (s, H-15), 1.85 (s, H-16), 1.62 (s, H-17), and two carbonyl groups at δ_C_ 198.3 (C-2), 208.1 (C-14). The coupling patterns in ^1^H-NMR spectrum and the correlations in ^1^H-^1^H COSY spectrum showed the presence of three spin systems of C-3-C-4-C-5, C-7-C-8-C-9, and C-11-C-12-C-13. In the HMBC spectrum the correlations of H-1, H-3, H-4/C-1, H-4, H-8/C-6, H-5/C-7, H-8, H-12/C-10, H-11/C-9, and H-12, H-14, H-15/C-14 clarified the connections of the two terminal methyl groups (C-1, 15), the two carbonyl groups and the three spin systems, suggesting a linear structure of pentadecatrien-2,14-dione. The HMBC correlations of H-5, H-7/C-16, H-16/C-5, C-7, H-9, H-11/C-17, and H-17/C-9, C-10, C-11 allowed the attachments of C-16 to C-6 and C-17 to C-10, which also further supported the presence of a pentadecatrien-2,14-dione moiety. 

A 3*E* geometry was assigned via its large ^3^*J*_3,4_ coupling constant (16.5 Hz). The NOESY correlations of H-5/H-7 and H-11/H-17 revealed that the geometries of the C-5 and C-10 olefins in **2** were 5*E* and 10*Z*. Compound **2** was characterized as (3*E*,5*E*,10*Z*)-7-hydroxy-6,10-dimethyl-pentadecatrien-2,14-dione. The optical rotation of **2** that is close to 0 suggested **2** is a pair of enantiomers, which was proved by its separation on HPLC using a chiral chromatography column. 

Compound **3** was obtained as a yellow oil. The molecular formula, C_21_H_24_O_5_, was established from its proton adduct ion in the HRESIMS (357.16873 [M + H]^+^, calcd. 357.1702), which was also supported by NMR data. Analysis of the ^1^H-NMR spectrum (Table 1) of **3** revealed the presence of a set of ABX system aromatic protons at δ_H_ 6.40 (d, *J* = 2.4 Hz, H-3′), 6.42 (dd, *J* = 8.4, 2.4 Hz, H-5′), and 7.25 (d, *J* = 8.4 Hz, H-6′), two *ortho*-coupled doublet aromatic protons at δ_H_ 6.79 (d, *J* = 8.4 Hz, H-5) and 6.30 (d, *J* = 8.4 Hz, H-6), and a set of aliphatic proton signals at δ_H_ 5.27 (dd, *J* = 9.6, 1.8 Hz, H-2), 2.20 (m, H-3a), 1.85 (m, H-3b), 2.86 (m, H-4a), 2.63 (m,H-4b), suggesting a flavan skeleton for **3**, which was consistent with the ^13^C-NMR data. The proton signals at δ_H_ 3.73 (dd, *J* = 7.2, 6.0 Hz, H-3″), 2.92 (dd, *J* = 17.4, 7.8 Hz, H-4″a), 2.54 (dd, *J* = 17.4, 5.4 Hz, H-4″b), 1.31 (s, 3H) and 1.22 (s, 3H), in combination with ^13^C-NMR signals at δ_C_ at 77.4 (C-2′′′), 70.5 (C-3′′′), 27.3 (C-4′′′), and 25.8, 20.8 (-Me) showed the presence of a 3″-hydroxyprenyl residue forming a furan ring with a hydroxyl group. The correlations of H-3″, H-4″a, H-4″b/C-8 confirmed the isoprenyl substituent was located at C-8, cyclizing onto 7-hydroxyl group. Accordingly, the structure of **3** was assigned as shown in Figure 1.

The optical rotation of **3** that was close to 0, suggesting **3** is a pair of enantiomers, which was proved by the separation on HPLC using a chiral chromatography column.

Compound **4** was obtained as a yellow oil. The IR spectrum of **4** showed the presence of hydroxy (3347 cm^−1^), carbonyl (1726 cm^−1^) and aromatic (1606 and 1466 cm^−1^) functional groups. The molecular formula, C_25_H_30_O_13_, was determined from its sodium adduct ion in the HRESIMS (561.1583 [M + Na]^+^, calcd. 561.1579) and also supported by the NMR spectroscopic data. The ^1^H-NMR (Table 1) spectrum showed signals attributable to a monosubstituted aromatic ring at δ_H_ 7.47 (2H, m, H-2′, 6′) and 7.36 (3H, m, H-3′, 4′, 5′), and an oxymethylene group at δ_H_ 5.33 (d, *J* = 12.6 Hz, H-7′a) and 5.22 (d, *J* = 12.6 Hz, H-7′b), revealing the presence of a benzyl group in combination with the correlations of H-7′/C-2′,6′ in HMBC spectrum. An ABC spin system attributed to anomeric protons at δ_H_ 6.65 (d, *J* = 8.4 Hz, H-3), 7.18 (dd, *J* = 8.4 Hz, H-4), and 6.55 (d, *J* = 8.4 Hz, H-5). The correlations of H-3, H-5/C-7 (δ_C_ 165.8) in HMBC spectrum showed the presence of a 2,6-bisubstituted benzoyl moiety. Together with the coupling patterns of oxymethylene and oxymethine protons resonating between δH 3.09 and 4.84 indicated the presence of a glucopyranosyl and a apiofuranosyl units. The correlations of H-7′/C-7, H-1″/C-2, H-1′′′/C-6″ in HMBC spectrum determined a moiety of apiofuranosyl(1→6)-glucopyranose was attached to C-2 of benzoyl moiety and the benzyl group was connected to C-7of the benzoyl moiety. A β-anomeric configuration for the glucosyl unit was assigned via its large ^3^*J*_1’’,2’’_ coupling constant (7.2 Hz). The β-configuration for apiofuranosyl unit was assigned via its ^3^*J*_1’’’,2’’’_ coupling constant (3.0 Hz) and the chemical shift of anomeric carbon (δ_C_ 109.4) [6,18]. The D-configurations of glucopyranosyl and a apiofuranosyl units were determined by gas chromatography (GC) analysis of the trimethylsilyl l-cysteine derivatives after acid hydrolysis of **4**. On the basis of the above data, **4** was characterized as benzyl 2-*O*-[β-d-apiofuranosyl(1→6)-β-d-glucopyranosyl]-2,6-dihydroxy-benzoate.

The known compound was identified as (9*R*)-hydroxyl-(10*E*,12*Z*,15*Z*)-octadecatrienoic acid (**5**) by NMR analysis and comparison with literature data [19].

### 2.2. Aldose Reductase Inhibitory Effects of ***2*** and **5** and Neuroprotective Effects of Compounds ***1**–**5***

The aldose reductase inhibitory and neuroprotective bioactivities of compounds **1**–**5** were assessed. Compounds **2** and **5** possessed inhibition activities against aldose reductase, with IC_50_ values of 4.33 μM and 6.0 μM compared with the potent AR inhibitor epalrestat (IC_50_ 1.88 × 10^−3^ μM) [20]. Compound **3** exhibited neuroprotective activity against PC12 cell damage induced by serum deprivation and **5** appeared to protect against PC12 cell damage caused by nicouline (Table 3), an assay extensively used in screening active agents for Parkinson’s disease [21,22,23,24]. Compounds **1**–**5** were evaluated for their cytotoxic activities against eight human cancer cell lines (human colon carcinoma cell line HCT-8, hepatocellular carcinoma cell line Bel-7402, human renal cell carcinoma cell line KETR3, Human cervical carcinoma cell line HELA, human gastric cancer cell line BGC-823, human ovarian carcinoma cell line A2780, human breast cancer cell line MCF-7, and human lung carcinoma cell line A549) by means of the MTT assay [25], using paclitaxel and 5-fluouracil as positive controls. Nevertheless, all the isolated compounds resulted to be inactive.

### 2.3. Discussion

We found it was helpful to subject the chloroform fraction of the ethanol extract to flash silica gel column chromatography repeatedly before isolation to remove pigments. The 80% MeOH-H_2_O mobile phase of Sephadex LH-20 column chromatography worked well for all kinds of structures in our study. We obtained two pairs of enantiomers, (**2a**,**2b**) and (**3a**,**3b**). Yang et al. reported an interesting phenomenon whereby the *R* and *S* configurations of C-2 of a similar flavan were interconvertible, which (**3a**,**3b**) may be subject to [5]. In addition, considering the wide range of examples that the isomers of enantiomers showed differences in pharmacological processes, further separation and research of the two pairs of enantiomers is needed.

Polyphenols from *Morus* plants have indicated extensive antioxidative activities, especially the kind of Diels-Alder type adducts [8,26]. Oxidative stress played a key role in neurodegenerative diseases, which implied the potential of polyphenols from *Morus* plants against neurodegenerative disorders [27]. Unfortunately, most of the previous studies on *Morus* polyphenols had been focused on their anti-oxidant properties. More efforts are suggested to explore the neuroprotective action of constituents of *Morus* plants.

## 3. Materials and Methods

### 3.1. Plant Material

The leaves of *Morus alba* L. were collected in the Anding Mulberry Garden (Beijing, China), in July 2011, and identified by Prof. Lin Ma (Institute of Materia Medica, Chinese Academy of Medical Sciences & Peking Union Medical College, Beijing, China). A voucher specimen (No. ID-S-2543) has been deposited at the Herbarium of Institude of Materia Medica, Chinese Academy of Medical Sciences & Peking Union Medical College.

### 3.2. General Experimental Procedures

Optical rotations were measured with a P-2000 polarimeter (Jasco, Tokyo, Japan) and UV spectra with a Jasco V-650 spectrophotometer. ECD spectra were measured on a Jasco J-815 spectrometer. IR spectra were recorded on a model 5700 spectrometer (Nicolet, Madison, SD, USA) by an FT-IR microscope transmission method. NMR measurements were performed using VNS-600 (Varian Medical Systems, Inc., Palo Alto, CA, USA), Mercury-300 (Varian Medical Systems, Inc., Palo Alto, CA, USA), Bruker-AV-III-500 (Bruker Corporation, Karlsruhe, Germany), and Inova-500 (Varian Medical Systems, Inc., Palo Alto, CA, USA) spectrometers. ESIMS was performed on Agilent 1100 Series LC/MSD Trap SL mass spectrometer and HRESIMS data were obtained using an Agilent 6520 Accurate-Mass Q-TOF LC/MS (Agilent Technologies, Ltd., Santa Clara, CA, USA). Gas chromatography (GC) was operated on Agilent 7890A system. HPLC was performed on a Lumtech instrument (Lumiere Tech Ltd. Beijing, China) equipped with a 500 ELSD detector (Alltech, Deerfield, IL, USA) and a YMC-Pack ODS-A column (250 × 20 mm, 5 μm, YMC, Tokyo, Japan). Silica gel (200−300 mesh, Qingdao Marine Chemical Factory, Qingdao, China), Sephadex LH-20 (GE), and ODS (50 μm, YMC) were used for column chromatography. TLC was carried out with GF254 plates (Qingdao Marine Chemical Factory). Spots were visualized by spraying with 10% H_2_SO_4_ in EtOH followed by heating.

### 3.3. Cell Lines, Chemicals and Biochemicals

PC12 cells (adrenal gland; pheochromocytoma) were purchased from the American Type Culture Collection (Manassas, VA, USA). Dimethyl sulphoxide (DMSO), nicouline, more commonly known as rotenone, and 3-(3,4-dimehylthiazol-2-yl)-2,5-diphenyl-tetrazolium bromide (MTT) were obtained from Sigma (St. Louis, MO, USA). Dulbecco’s Modified Eagle’s Medium (DMEM), fetal bovine serum (FBS), and horse serum were purchased from Gibco BRL (New York, NY, USA). Epelrestat was purchased from Dayin Marine Bio-Pharmaceutical Co., Ltd. (Rongcheng, Shandong, China). NADPH-Na4, paclitaxel and 5-fluorouracil were purchased from Sigma-Aldrich (Beijing, China). All other chemicals were of analytical grade and were commercially available.

### 3.4. Extraction and Isolation

Air-dried leaves of *Morus alba* L. (30 kg) were exhaustively extracted with 95% aqueous EtOH (3 × 100 L, 2 h) at reflux. The combined extracts were concentrated under reduced pressure to dryness. The residue (2.9 kg) was subjected to column chromatography on silica gel and eluted with petroleum ether, chloroform, acetone and methanol. The acetone residue (423 g) was subjected to D101 macroporous resins column chromatography by a gradient elution with EtOH/H_2_O (0:100, 30:70, 60:40, 95:5) to yield four fractions (fractions A–D). The separation of fraction B (120 g) was carried out on silica gel column chromatography eluted with CHCl_3_/MeOH (10:1–3:1) to provide three subfractions B1–B3. Subfraction B2 (80 g) was further purified by MPLC (ODS, 50 μm, YMC) and eluted with 15, 35, 55, 75 and 100% MeOH−H_2_O, to afford 40 subfractions. Fraction B2-18 (42 mg) was purified by preparative HPLC using 20% MeCN−H_2_O (8 mL/min) as the mobile phase to yield compound **1** (4 mg) and fraction B2-20 (20 mg) was purified by preparative HPLC using 25% MeCN−H_2_O (8 mL/min) to yield compound **4** (18 mg). The chloroform residue (355 g) was subjected to silica gel column chromatography by a gradient elution with petroleum ether/acetone (100:0, 95:5, 90:10, 80:20, 70:30, 60:40) to yield six fractions (fractions E–M). The separation of fraction M (38 g) was carried out by MPLC (ODS, 50 μm, YMC) and eluted with 5, 15, 35, 55, 75, and 100% MeOH−H_2_O, to afford 25 subfractions. Fraction M-7 (100 mg) was purified by preparative HPLC using 20% MeOH−H_2_O (8 mL/min) as the mobile phase to yield compound **5** (45 mg). The separation of fraction N (20 g) was carried out by MPLC (ODS, 50 μm, YMC) and eluted with 10, 30, 55, 75, and 100% MeOH−H_2_O, to afford 28 subfractions. Fraction N-6 (50 mg) was purified by preparative HPLC using 30% MeOH−H_2_O (8 mL/min) as the mobile phase to yield compound **2** (10 mg). The separation of fraction g (139 g) was carried out by MPLC (ODS, 50 μm, YMC) and eluted with 15, 35, 55, 75, 85, and 100% MeOH-H2O, to afford 38 subfractions. Fraction G-10 (385 mg) was subjected to fractionation using Sephadex LH-20 column chromatography (80% MeOH-H_2_O) to provide 40 subfractions. Fraction G-10-18 (42 mg) was purified by the preparative HPLC using 35% MeCN−H_2_O (8 mL/min) as the mobile phase to yield compound **3** (15 mg).

### 3.5. Characterization

*Moralsin* (**1**): white powder; ${\left[\alpha \right]}_{D}^{20}$ −72 (*c* 0.19, MeOH); UV(MeOH): λ_max_ (log ε) 228 (2.10) nm; IR ν_max_ 3402, 2957, 1760, 1606, 1080 cm^−1^; ^1^H-NMR (DMSO-*d*_6_, 500 MHz) and ^13^C-NMR (DMSO-*d*_6_, 125 MHz) see Table 2; positive-ion HRESIMS *m*/*z* 433.1826 [M + Na]^+^ (calcd. 433.1833).

*(3E,5E,10Z)-7-Hydroxy-6,10-dimethyl-pentadecatrien-2,14-dione* (**2**): yellow oil; UV(MeOH): λ_max_ (log ε) 207 (4.31) nm, 247 (0.11) nm; IR ν_max_ 3347, 2926, 1726, 1606, 1466, 1051, 1025 cm^−1^; ^1^H-NMR (DMSO-*d*_6_, 500 MHz) and ^13^C-NMR (DMSO-*d*_6_, 125 MHz) see Table 1 and Table 2; positive-ion HRESIMS *m*/*z* 561.1583 [M + Na]^+^ (calcd. 561.1579).

*2′-Hydroxy-4′-methoxyl-2H-(2′′′, 2′′′-dimethyl-3′′′-hydroxy)-pyran-(5′′′,6′′′:8,7)-flavane* (**3**): yellow oil; UV(MeOH): λ_max_ (logε) 206 (3.90) nm, 280 (2.80) nm; IR ν_max_ 3372, 1718, 1602 cm^−1^; ^1^H-NMR (MeOH-*d*_4_, 600 MHz) Table 1; ^13^C-NMR (MeOH-*d*_4_, 150 MHz) Table 2; positive-ion HRESIMS *m*/*z* 357.16873 [M + H]^+^ (calcd. for C_21_H_25_O_5_, 357.1702).

*Benzyl 2-O-[β-d-apiofuranosyl(1→6)-β-d-glucopyranosyl]-2,6-dihydroxybenzoate* (**4**): yellow oil; UV(MeOH): λ_max_ (log ε) 207 (4.32) nm, 247 (0.1) nm; IR ν_max_ 3347, 2926, 1726, 1606, 1466, 1051, 1025 cm^−1^; ^1^H-NMR (DMSO-*d*_6_, 300 MHz) and ^13^C-NMR (DMSO-*d*_6_, 125 MHz) see Table 1 and Table 2; positive-ion HRESIMS *m*/*z* 561.1583 [M + Na]^+^ (calcd. 561.1579).

*(9R)-Hydroxy-(10E,12Z,15Z)-octadecatrienoic acid* (**5**): Yellow powder, ${\left[\alpha \right]}_{D}^{20}$ −3.04° (0.58 CHCl_3_); ESIMS *m*/*z* 317.2 [M + Na]^+^, ^1^H-NMR (DMSO-*d*_6_, 500 MHz) δ: 2.18 (2H, t, 9.6), 1.47 (2H, m, H-3), 1.38 (2H, m, H-8), 2.04 (2H, m, H-17), 0.92 (3H, t, *J* = 9.6 Hz, H-18), 3.97 (1H, m, H-9), 5.66 (1H, dd, *J* = 15.5, 6.0 Hz, H-10), 6.44 (1H, dd, *J* = 15.5, 11.5 Hz, H-11), 5.26-5.40 (3H, m, H-12, 15, 16), 5.96 (1H, t, *J* = 11.0 Hz, H-13), 2.88 (2H, dd, *J* = 11.0, 7.5, Hz, H-14), 1.24 (6H, m, H-4-6). ^13^C-NMR (DMSO-*d*_6_, 125MHz) δ: 174.5 (C-1), 138.6 (C-10), 131.7 (C-16), 128.7 (C-13), 128.3 (C-12), 126.7 (C-15), 123. 5 (C-11), 70.44 (C-9), 37.2 (C-8), 33.7 (C-2), 28.5, 28.8, 28.9 (C-4-6), 25.5 (C-14), 24.9 (C-7), 24.5 (C- 3), 20.0 (C-17), 14.1 (C-18).

### 3.6. Acid Hydrolysis of the Saponins and Determination of the Absolute Configuration of the Monosaccharides

Compound **1** (2 mg) was hydrolyzed in 2 M HCl/H_2_O at 80 °C for 2 h. The residue was reacted sequentially with l-cysteine methyl ester hydrochloride and N-trimethylsilylimidazole. The resulting monosaccharide N-trimethylsilylimidazole derivatives were analyzed by GC. d-Glucose was confirmed by comparison of the retention time of the derivatives with those of authentic sugars derivatized in a similar way, which showed retention times of 27.93 min. The constituent sugars of compounds **4** were identified by the same method as **1**. Retention times of authentic samples were detected at 17.87 min (d-apiofuranose) and at 27.93 (d-glucose). The reaction and GC conditions were as described in the literature [28].

### 3.7. Aldose Reductase Assay 

The assay was operated in 96 well culture plate. A 100 μL mixture that contained 10 mM DL-glyceraldehyde, 0.16 mM NADPH-Na4 and aldose reductase in 0.1 M sodium phosphate buffer (pH 6.2), with or without test compounds was prepared at 0 °C. Appropriate blank were employed for corrections. The assay mixture was incubated at 25 °C. After 10 min of incubation, the plate was immediately cooled at −20 °C for 5 min to stop the reaction. The change in the absorbance at 340 nm due to NADPH oxidation was measured in a plate reader [29].

### 3.8. Neuroprotection Bioassays 

The PC12 cells were cultured in DMEM medium supplemented with 5% horse serum and 5% fetal bovine serum. Then, 100 μL of cells with an initial density of 5 × 10^4^ cells/mL was seeded in each well of a poly-L-lysine-coated, 96-well culture plate and precultured for 24 h. The medium was then replaced by different fresh medium including the control (complete medium), the model (complete medium with 4 μM rotenone or serum free medium), and the sample (the test compounds with different drug concentrations, 10, 1, and 0.1 μM, were added to the aforementioned model medium), and the cells were cultured for 48 h. Then, 10 μL of MTT (5 mg/mL) was added to each well. After incubation for 4 h, the medium was removed, and 150 μL of DMSO was added to dissolve the formazan crystals. The optical density (OD) of the PC12 cells was measured on a microplate reader at 570 nm [30]. 

## 4. Conclusions

The plants of genus *Morus* have been extensively investigated for their medicinal constituents and a series of unique structures were characterized [31,32,33,34,35,36,37]. This studied focused on the fractions that had been rarely researched before and generated four new structures with aldose reductase inhibitory or neuroprotective activities. The known molecule **5** was isolated for the first time from the genus *Morus* and its neuroprotective activity reported for the first time. The compounds reported here provide new potential aldose reductase inhibitory or neuroprotective agents for further research.

## Figures and Tables

**Table 1:** ^1^H-NMR Spectroscopic Data of Compounds **1**–**4**^a^.

1		2		3		4	
Position	δ_H_ (*J* in Hz)	Position	δ_H_ (*J* in Hz)	Position	δ_H_ (*J* in Hz)	Position	δ_H_ (*J* in Hz)
2α	1.86, (overlapped)	1	2.25, s	2	5.27, dd, (9.6, 1.8)	3	6.65, d, (8.4)
2β	1.60, dd, (15.0, 6.5)	3	6.09, d, (16.5)	3a	2.20, m	4	7.18, dd, (8.4)
3	4.41, m	4	7.45, dd, (16.5, 11.5)	3b	1.85, m	5	6.55, d, (8.4)
4	6.82, t, (3.0)	5	6.23, d, (11.5)	4a	2.86, m	2′, 6′	7.47, m
6	2.57, m	7	3.93, t, (6.5)	4b	2.63, m	3′, 5′	7.36, m
7	5.12, t, (9.3)	8	1.47, m	5	6.79, d, (8.4)	4′	7.36, m
8	5.66, d, (9.3)	9	1.97, t, (7.5)	6	6.30, d, (8.4)	7′a	5.33, d, (12.6)
10	6.44, dd, (17.5, 9.0)	11	5.03, t, (7.0)	3′	6.40, d, (2.4)	7′b	5.22, d, (12.6)
11a	5.37, d, (17.5)	12	2.12, dd, (7.5, 7.0)	5′	6.42, dd, (8.4, 2.4)	1″	4.84, d, (7.2)
11b	5.20, d, (10.5)	13	2.42, t, (7.5)	6′	7.25, d, (8.4)	2″	3.21, m
12	0.90, s	15	1.06, s	3′′	3.73, dd, (7.2, 6.0)	3″	3.49, m
13	0.87, 3H, s	16	1.85 (3H, s)	4′′a	2.92, dd, (17.4, 7.8)	4″	3.09, t, (8.7)
15	1.88, s	17	1.62 (3H, s)	4′′b	2.54, dd, (17.4, 5.4)	5″	3.25, m
1′	4.35, d, (8.0)			5′′	1.22, s ^b^	6″a	3.86, d, (8.1)
2′	2.91, t, (8.0)			6′′	1.31, s ^b^	6″b	3.44, m
3′	3.15, m			-OMe	3.75, s	1′′′	4.83, d, (3.0)
4′	3.03, t, (8.0)					2′′′	3.75, d, (3.0)
5′	3.15, m					4′′′a	3.89 (1H, d, 9.0)
6′a	3.68, dd, (10.5, 1.0)					4′′′a	3.59 (1H. d, 9.0)
6′b	3.44, dd, (10.5, 6.0)					5′′′	3.35 (1H, m)

^a 1^H–NMR data (δ) were measured at 500 MHz in DMSO-*d*_6_ for **1**, **2**, **4**, and at 600 MHz in methanol-*d*_4_ for **3**. The assignments were based on HSQC and HMBC experiments; ^b^ Interchangeable.

**Table 2:** ^13^C-NMR Spectroscopic Data of Compounds **1**–**4**^a^.

1		2		3		4	
Position	δ_c_, Type	Position	δ_c_, Type	Position	δ_c_, Type	Position	δ_c_, Type
1	29.5, C	1	27.1, CH_3_	2	74.2, CH	1	120.0, C
2	43.2, CH_2_	2	198.3, CH	3	29.9, CH_2_	2	155.3, C
3	70.4, CH	3	129.9, CH	4	25.8, CH_2_	3	105.5 ^b^, CH
4	132.4, CH	4	139.4, CH	5	128.5, CH	4	131.0, CH
5	130.7, C	5	122.3, CH	6	109.7, CH	5	109.4 ^b^, CH
6	52.3, CH	6	153.1, C	7	153.0, C	6	155.4, C
7	76.8, CH	7	74.7, CH	8	109.3, C	7	165.8, C
8	129.8, CH	8	33.3, CH_2_	4a	114.4, C	1′	136.2, C
9	138.8, C	9	27.5, CH_2_	8a	154.5, C	2′, 6′	127.8, CH
10	140.0, CH	10	135.3, C	1′	122.4, C	3′, 5′	128.3, CH
11	115.6, CH_2_	11	124.0, CH	2′	156.1, C	4′	127.8, CH
12	21.0, CH_3_	12	21.8, CH_2_	3′	102.1, CH	7′	66.0, CH_2_
13	28.9, CH_3_	13	43.1, CH_2_	4′	161.4, C	1”	100.4, CH
14	168.8, C	14	208.1, C	5′	105.7, CH	2”	73.27, CH
15	12.3, CH_3_	15	29.8, CH_3_	6′	128.0, CH	3”	75.6, CH
1′	102.7, CH	16	13.4, CH_3_	2”	77.4, C	4”	70.0, CH
2′	73.5, CH	17	23.2, CH_3_	3”	70.5, CH	5”	76.8, CH
3′	76.8, CH			4”	27.3, CH_2_	6”	67.8, CH2
4′	70.1, CH			5′′	20.8	1′′′	109.4, CH
5′	76.9, CH			6′′	25.8	2′”	75.9, CH
6′	61.2, CH_2_ b 4.29, dd, (11.5, 6)			-OMe	55.7	3′′′	78.7, C
						4′′′	73.30, CH_2_
						5′′′	63.2, CH_2_

^a 1^H NMR data (δ) were measured at 125 MHz in DMSO-*d*_6_ for **1**, **2**, **4**, and at 150 MHz in methanol-*d*_4_ for **3**. The assignments were based on HSQC and HMBC experiments; ^b^ interchangeable.

**Table 3:** Neuroprotective Effects of Compounds **1**–**5** at concentration of 10^−5^ M (means ± SD, *n* = 6).

Sample	Serum Deprivation (%)	Nicouline 4 μM (%)
control	100.0 ± 3.7	100.0 ± 1.4
model	41.4 ± 3.8 ^###^	74.3 ± 1.4 ^###^
1	50.2 ± 12.7	78.6 ± 2.9
2	66.2 ± 12.6	78.4 ± 2.0 *
3	59.8 ± 2.7***	75.7 ± 3.0
4	50.9 ± 7.8	76.9 ± 3.6
5	70.2 ± 16.1	86.2 ± 7.6 ***

^###^*p* < 0.001 vs. control,* *p* < 0.05,** *p* < 0.001, *** *p* < 0.0001 vs. model.

## Supplementary Materials

The following are available online.
