# Supplementary material for: A Chemical Investigation of the Leaves of Morus alba L
Source: Molecules. 2018 Apr 26;23(5):1018. doi: 10.3390/molecules23051018 (PMC6102566; doi:10.3390/molecules23051018)
Supplement: Supplementary file 1 [file molecules-23-01018-s001.pdf]

Supplementary Materials

A Chemical Investigation of the Leaves of *Morus alba* L.

Xiao-yan Chen [1], Ting Zhang [2], Xin Wang [3], Mark T. Hamann [1], Jie Kang [4], De-quan Yu [4] and Ruo-yun Chen [4,*]

1 Department of Drug Discovery and Biomedical Sciences, College of Pharmacy, Medical University of South Carolina, Charleston, SC 29425, USA; chenxiaoyan8615@gmail.com or chenxi@musc.edu (X.-y.C.); hamannm@musc.edu (M.T.H.)
2 Institute of Medical Information & Library, Chinese Academy of Medical Sciences and Peking Union Medical College, Beijing 100020, China; brendatingting@126.com
3 Beijing Key Laboratory of Bioactive Substances and Function Foods, Beijing Union University, Beijing 100191, China; shtwangxin@buu.edu.cn
4 State Key Laboratory of Bioactive Substance and Function of Natural Medicines, Institute of Materia Medica, Chinese Academy of Medical Sciences and Peking Union Medical College, Beijing 100050, China; jiekang@imm.ac.cn (J.K.); dqyu@imm.ac.cn (D.-q.Y.)
* Correspondence: rych@imm.ac.cn; Tel.: +86-10-8316-1622

Content list:

		Pages			Pages
Key HMBC correlations	**1-4**	3-4	Compound **3**	UV	26
				IR	27
				^{1}H-NMR	28-30
				^{13}C-NMR	31
				^{1}H-^{1}H COSY	32
				HSQC	33
				HMBC	34
				MS	35
Compound **1**	UV	5	Compound **4**	UV	36
	IR	6		IR	37
	^{1}H-NMR	7-9		^{1}H-NMR	38
	ROESY	10		^{13}C-NMR	39
	^{13}C-NMR	11		DEPT	40
	^{1}H-^{1}H COSY	12		^{1}H-^{1}H COSY	41
	DEPT	13		HSQC	42
	HSQC	14		HMBC	43
	HMBC	15		MS	44
	MS	16			
	ECD	16			
Compound **2**	UV	17	Compound **5**	NMR data	45
	IR	18			
	^{1}H-NMR	19-20	Separation with chiral column	**2-3**	46
	^{13}C-NMR	21			
	^{1}H-^{1}H COSY	22			
	HSQC	23	GC	Standard monosaccharides	47
	HMBC	24		**1, 4**	
	MS	25			48

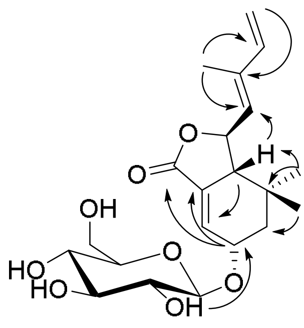

Figure S1. Key HMBC correlations (H → C) of compound **1**

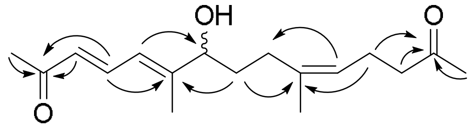

Figure S2. Key HMBC correlations (H→ C) of compound **2**

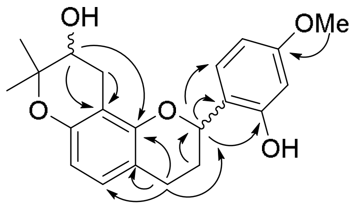

Figure S3. Key HMBC correlations (H→ C) of compound **3**

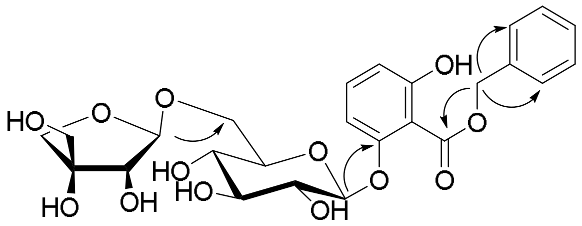

Figure S4. Key HMBC correlations (H→C) of compound **4**

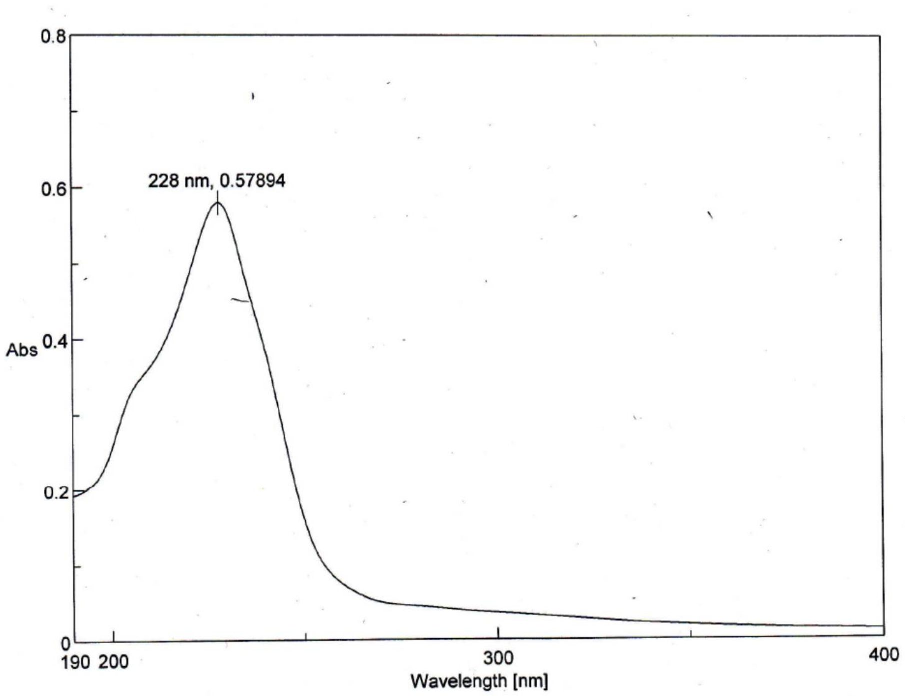

Figure S5. UV Spectrum of Compound **1.**

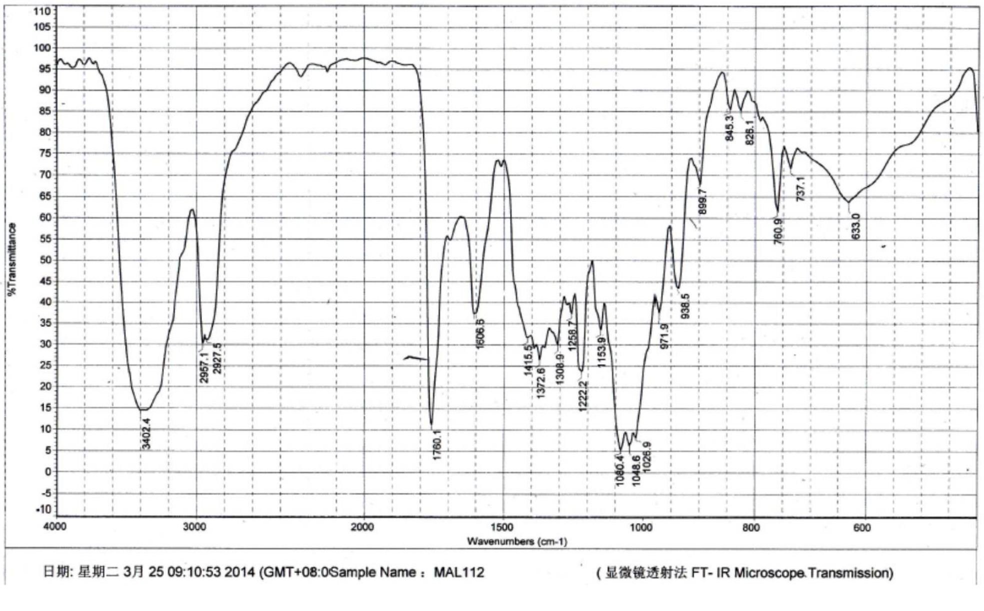

Figure S6. IR Spectrum of Compound **1**

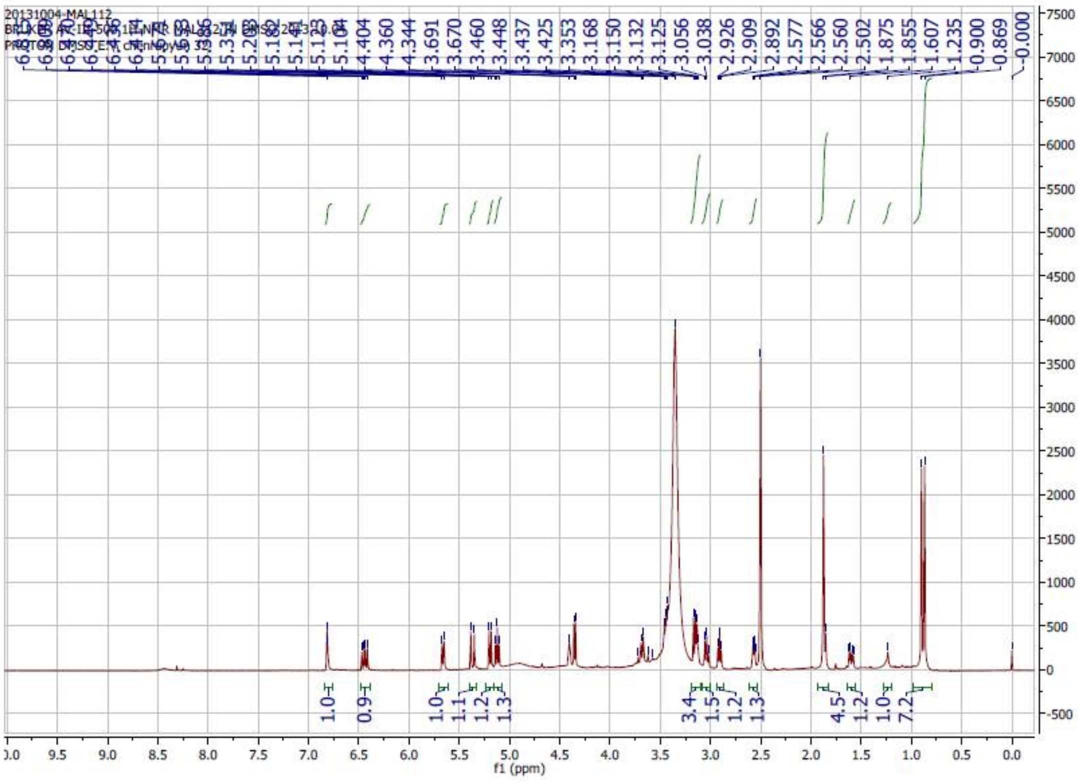

Figure S7.^{1}H-NMR Spectrum of Compound **1** (500 MHz, DMSO-d_6).

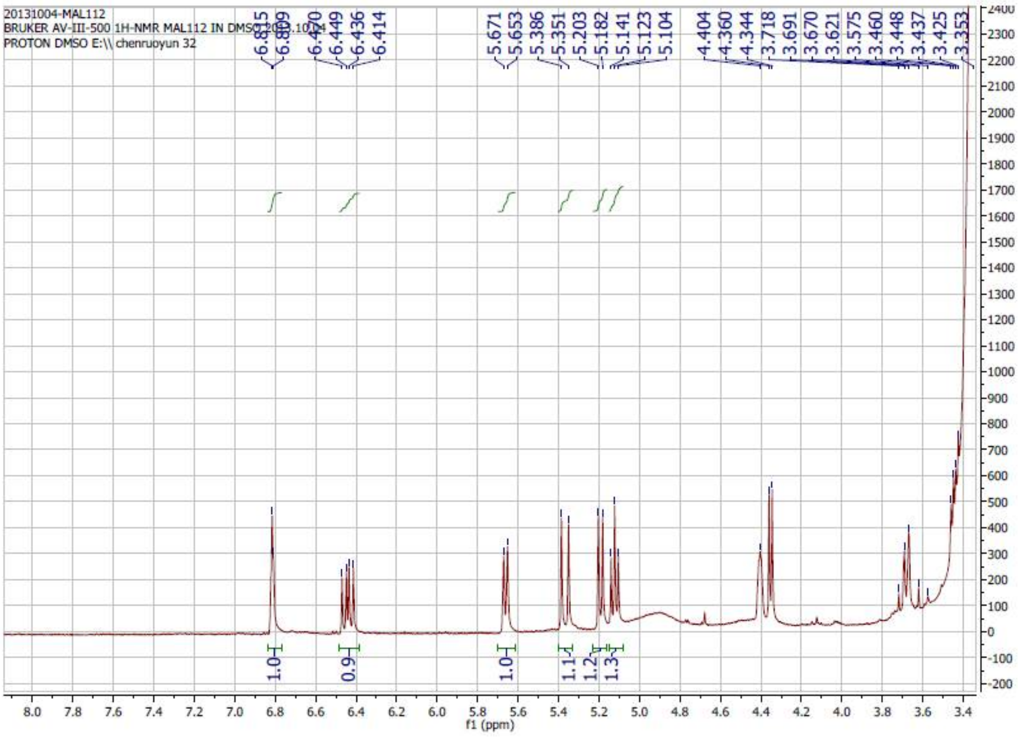

Figure S8. Enlarged ^{1}H-NMR Spectrum of Compound **1** (500 MHz, DMSO-d_6)

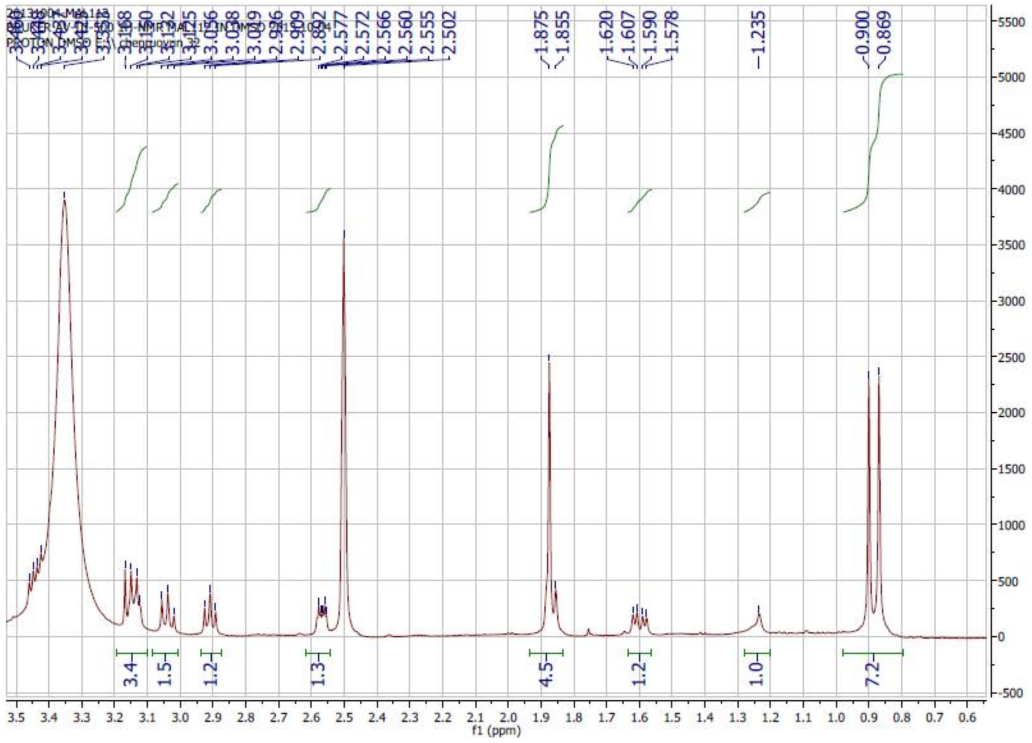

Figure S9. Enlarged ^{1}H-NMR Spectrum of Compound **1** (500 MHz, DMSO-d_6)

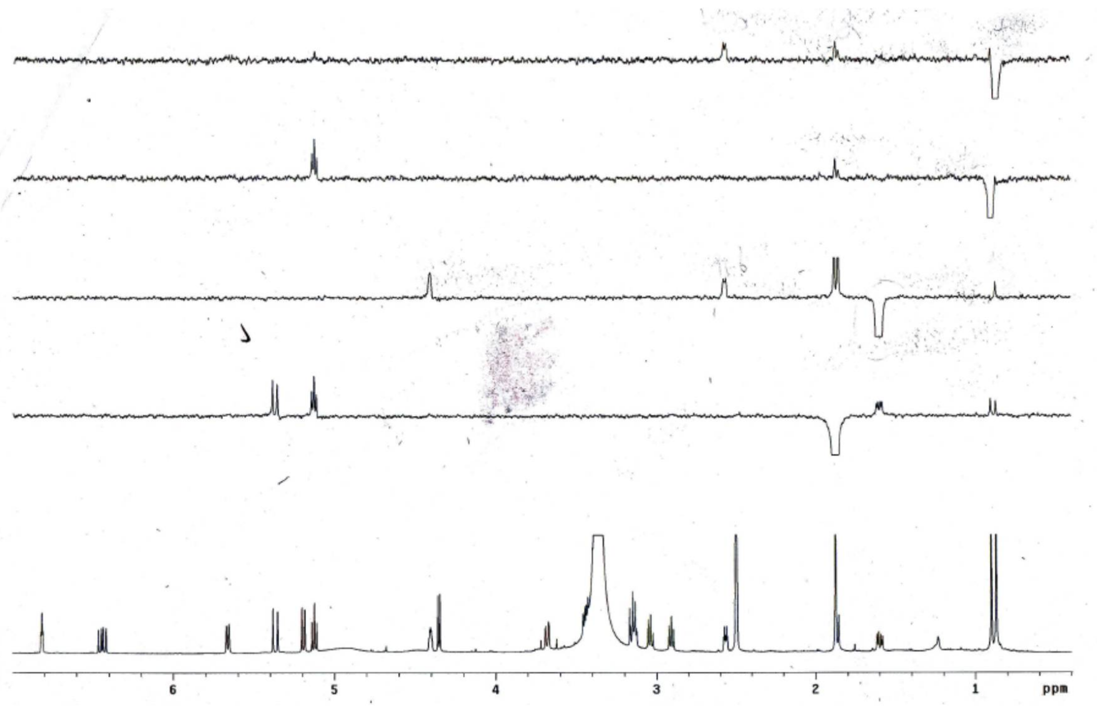

Figure S10. ROESY Spectrum of Compound **1** (DMSO-d_6, 600 MHz)

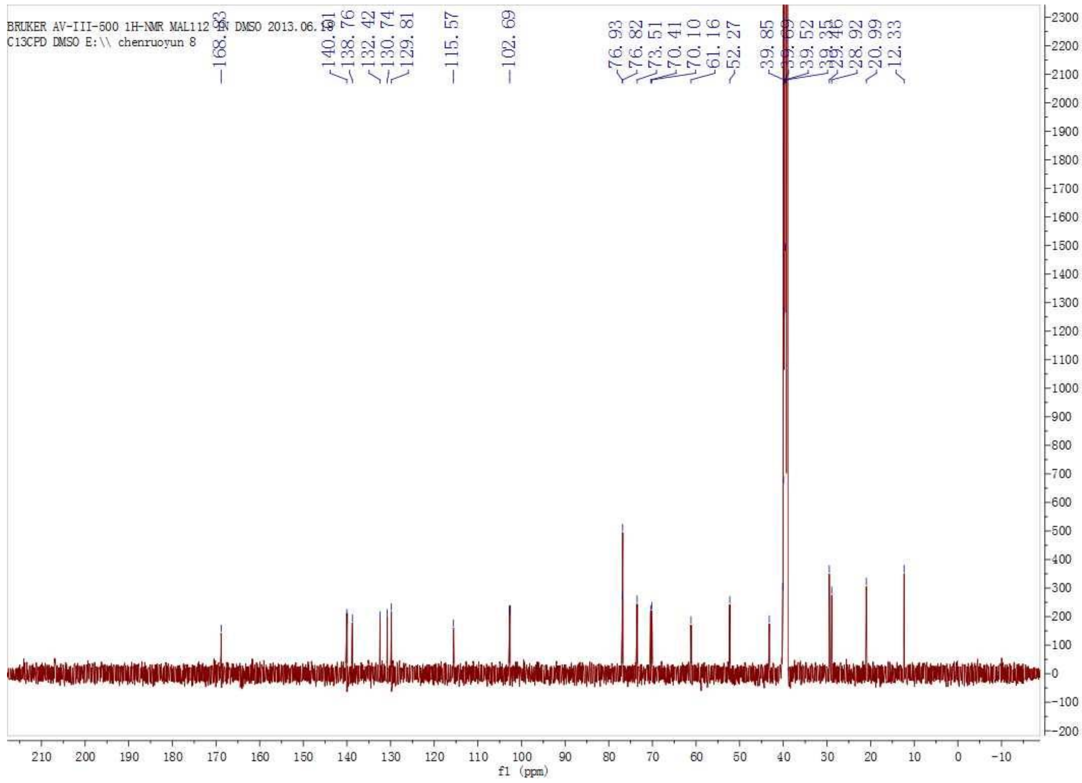

Figure S11. ^{13}C-NMR Spectrum of Compound **1** (125 MHz, DMSO-d_6)

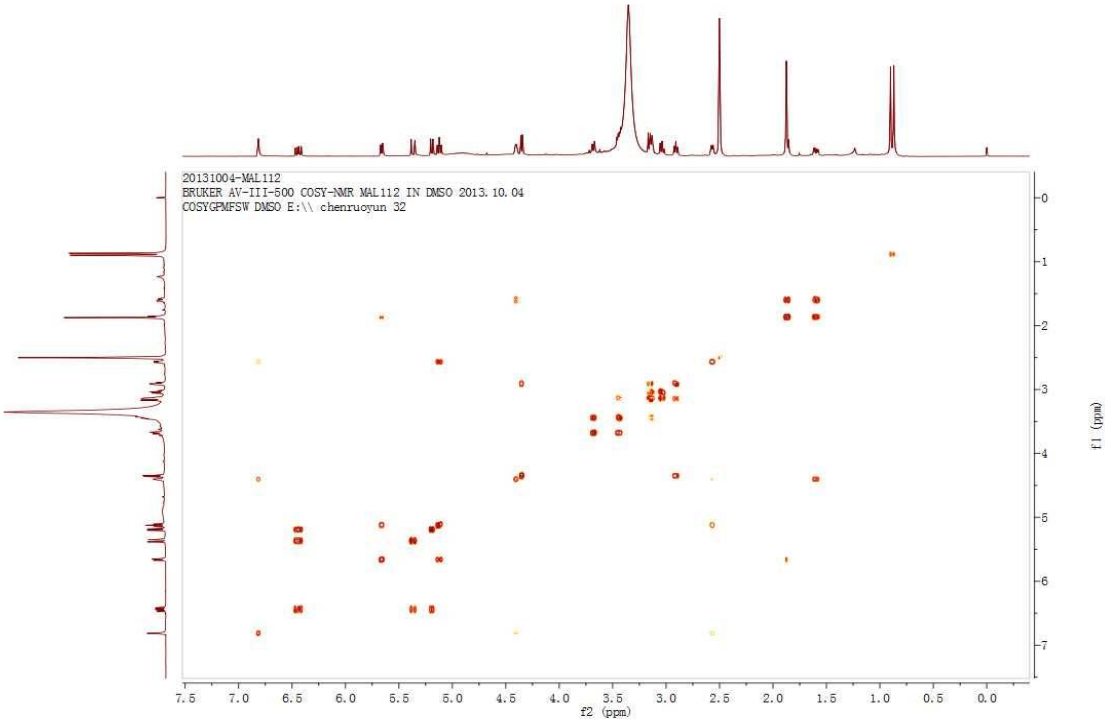

Figure S12. ^{1}H-^{1}H COSY Spectrum of Compound **1** (DMSO-d_6, 500 MHz)

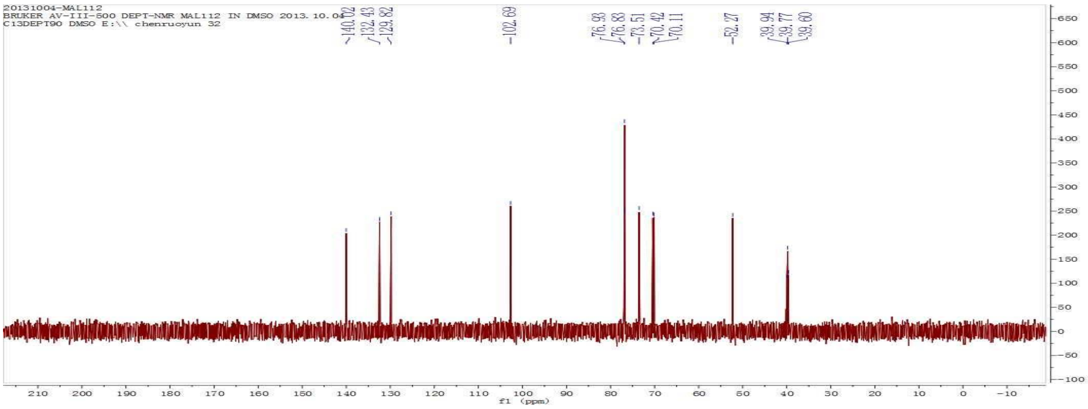

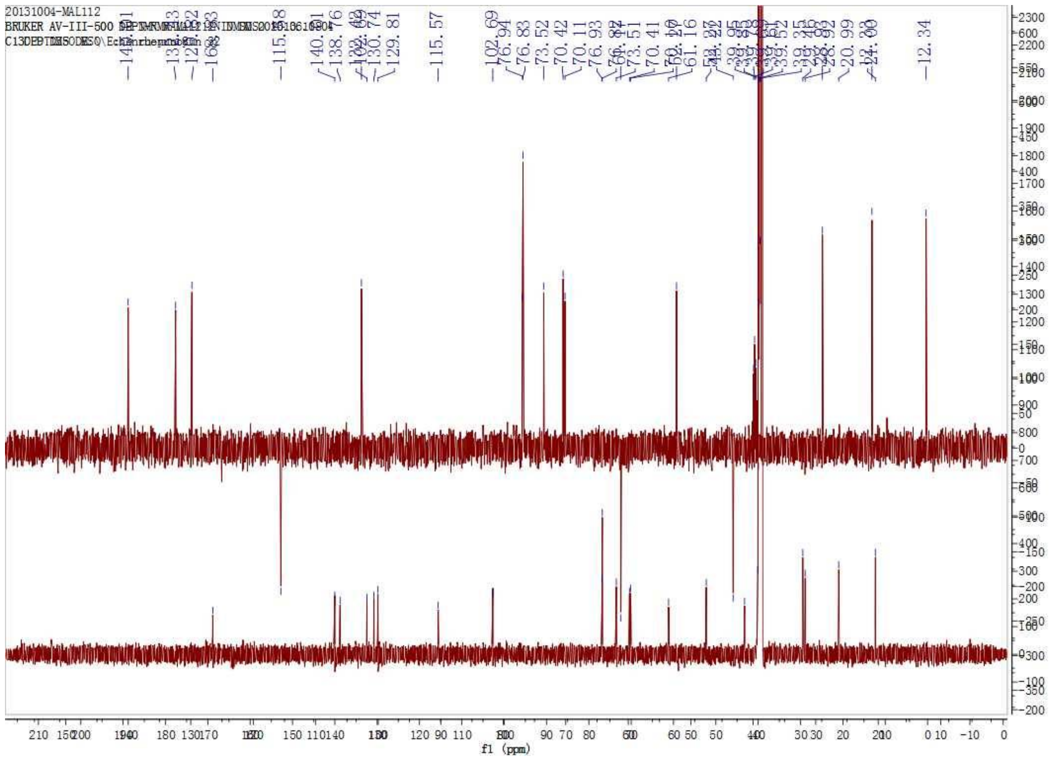

Figure S13. DEPT Spectrum of Compound **1** (DMSO-d_6, 125 MHz)

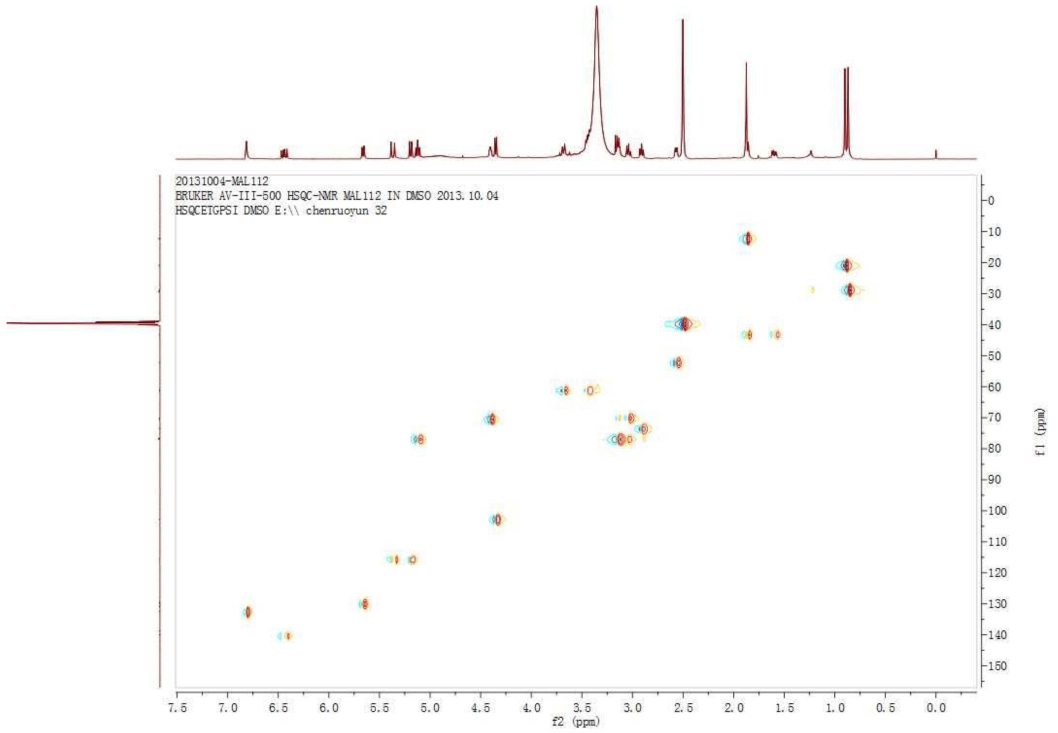

Figure S14. HSQC Spectrum of Compound **1** (500 MHz, DMSO-d_6).

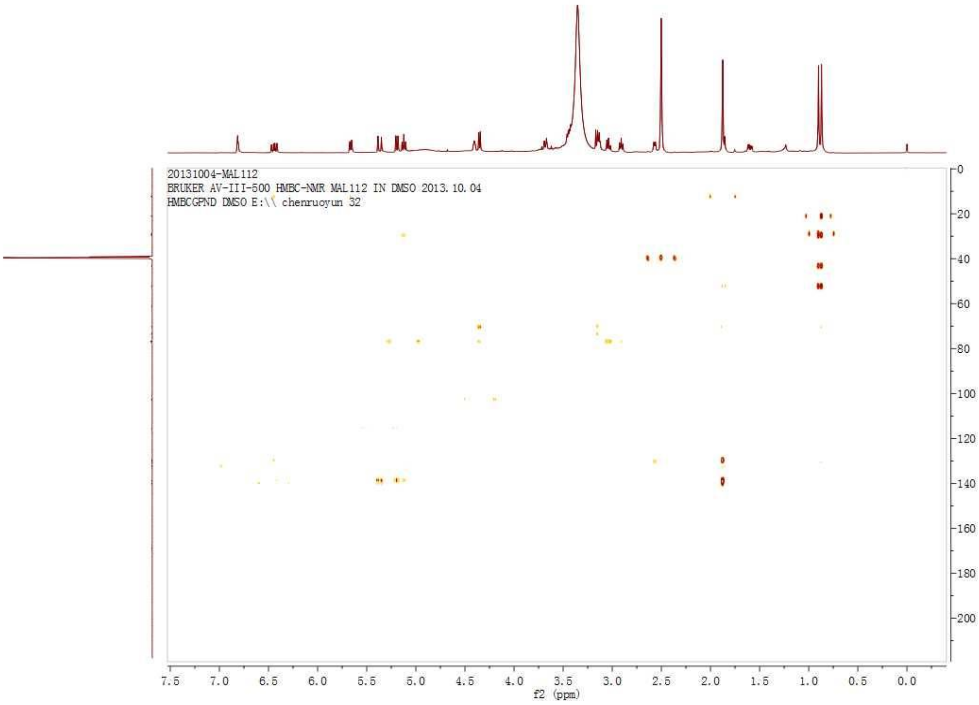

Figure S15. HMBC Spectrum of Compound **1** (500 MHz, DMSO-d_6)

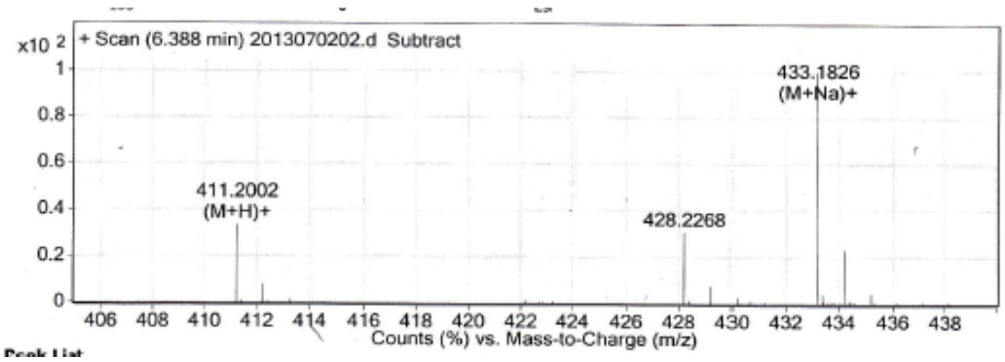

Figure S16. HR-ESIMS Spectrum of Compound **1**

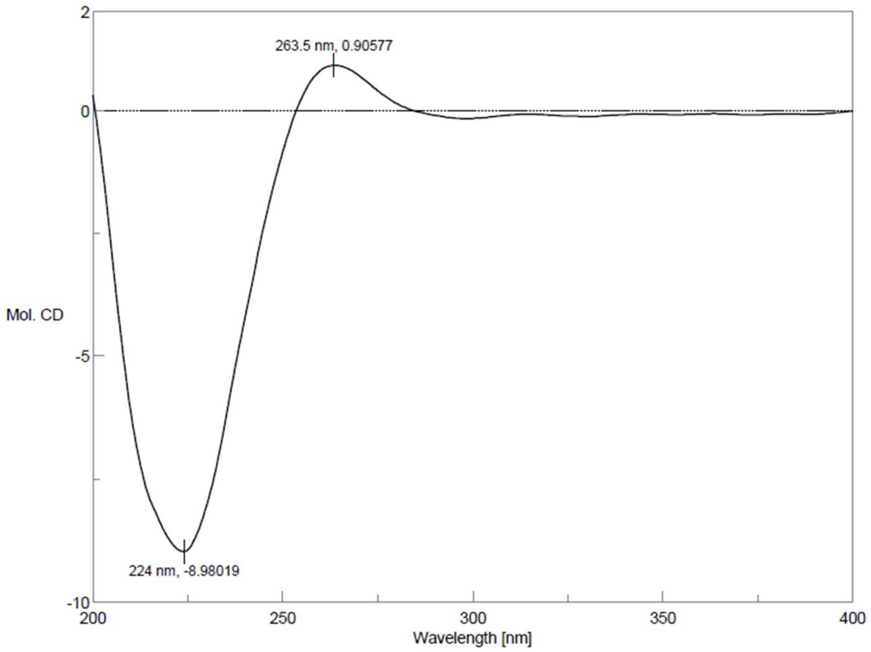

Figure S17. ECD Spectrum of Compound **1.**

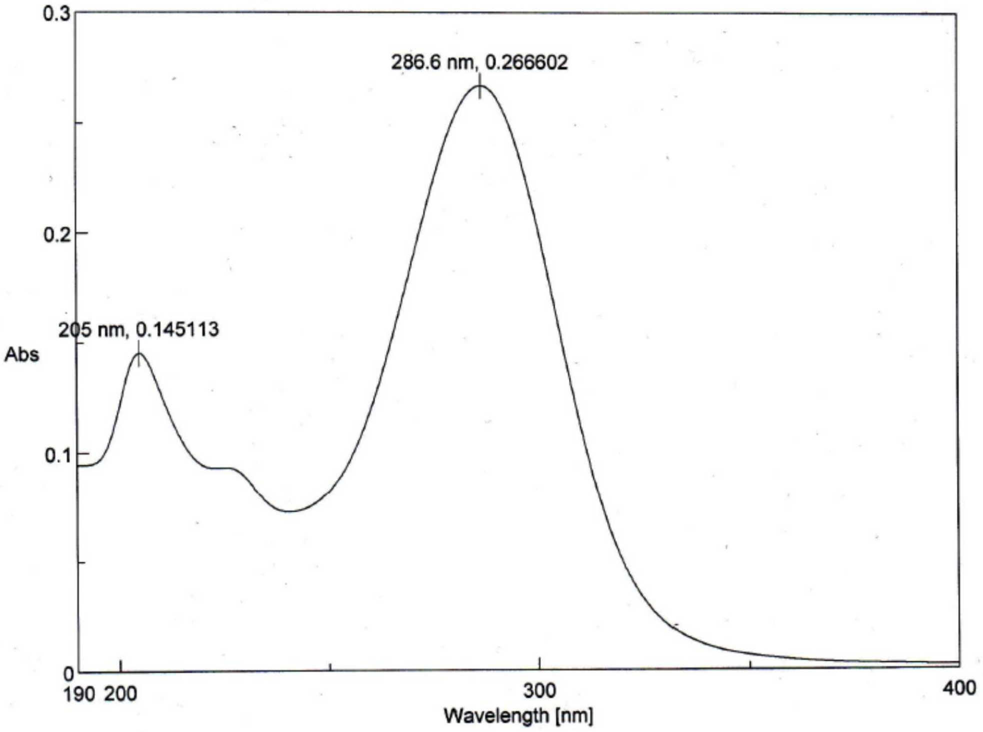

Figure S18. UV Spectrum of Compound **2.**

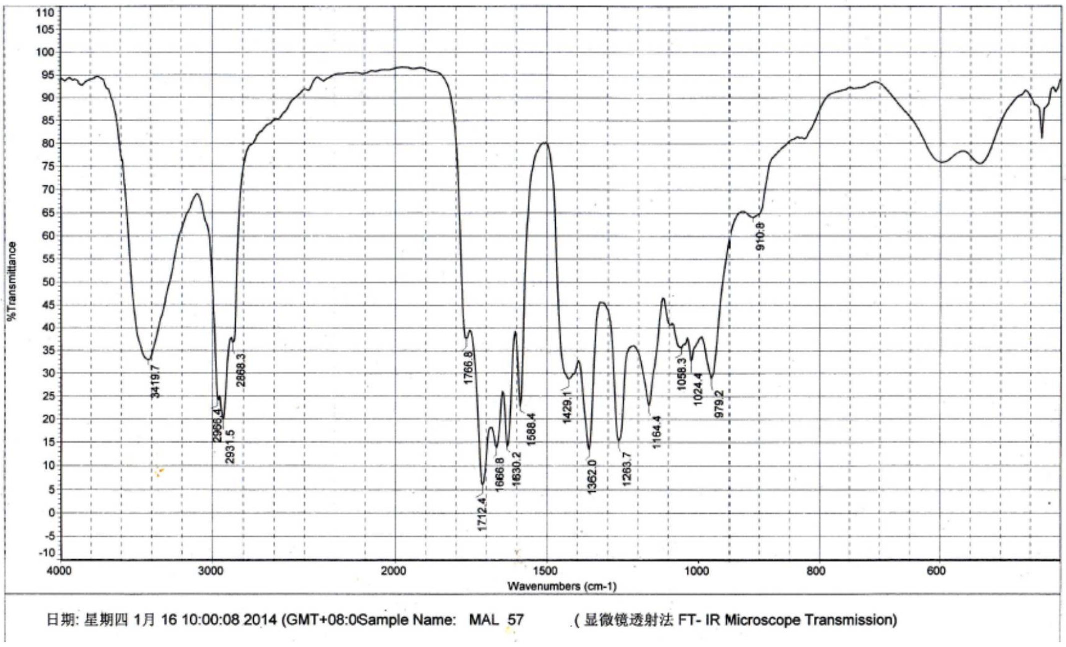

Figure S19. IR Spectrum of Compound **2.**

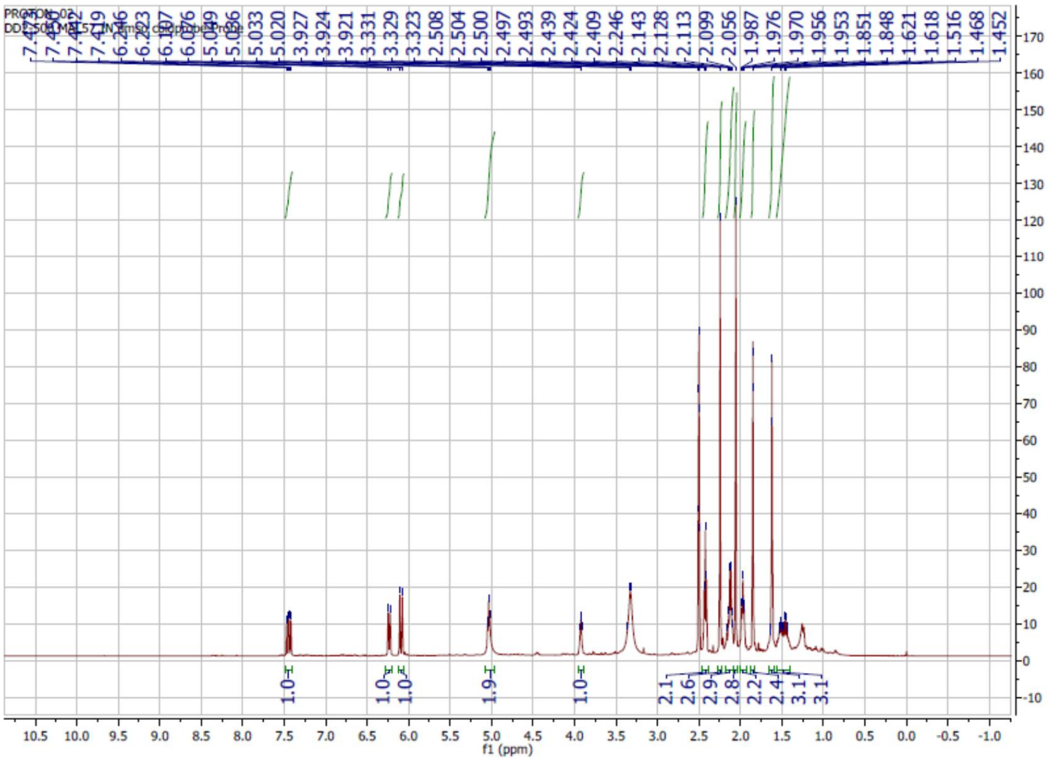

Figure S20. ^{1}H-NMR Spectrum of Compound **2** (500 MHz, DMSO-d_6).

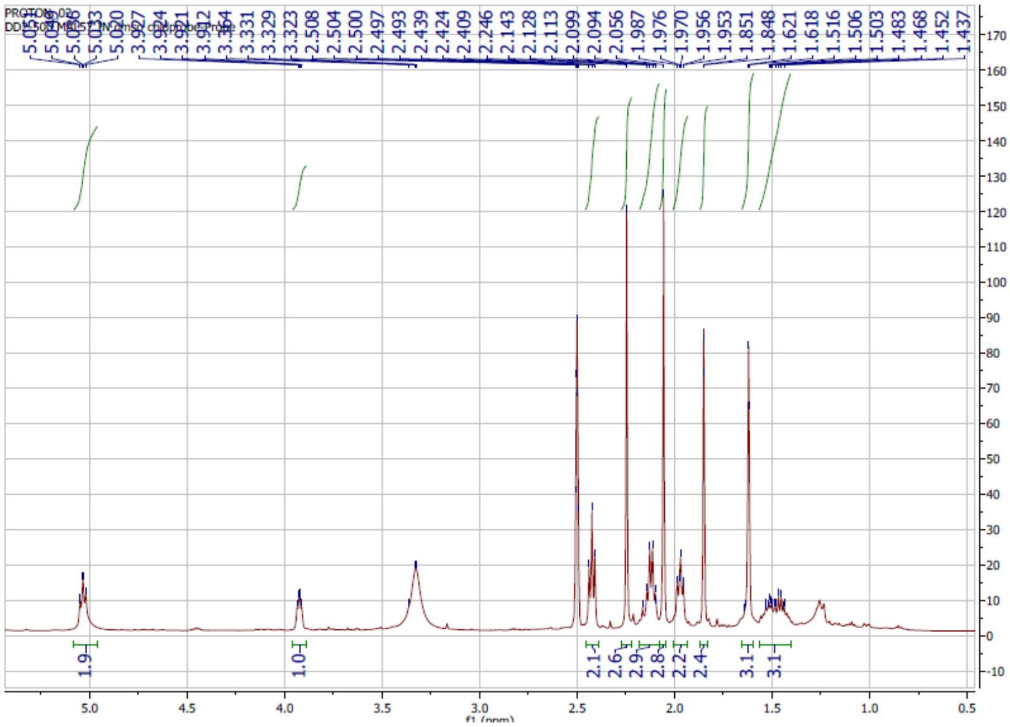

Figure S21. Enlarged ^{1}H-NMR Spectrum of Compound **2** (500 MHz, DMSO-d_6)

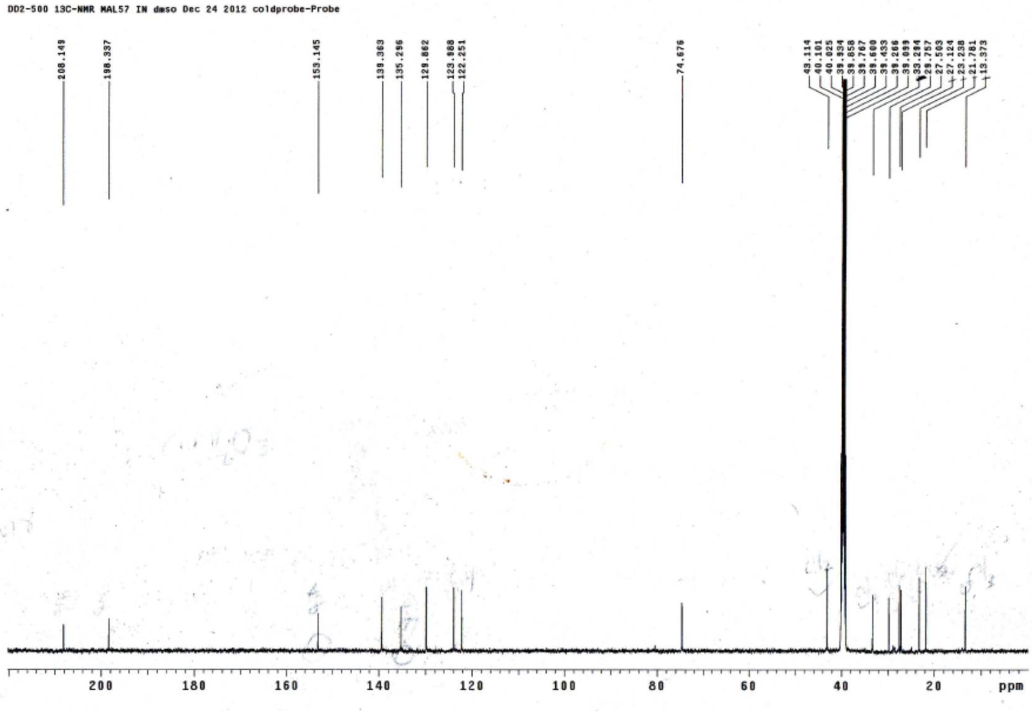

Figure S22. ^{13}C-NMR Spectrum of Compound **2** (125 MHz, DMSO-d_6)

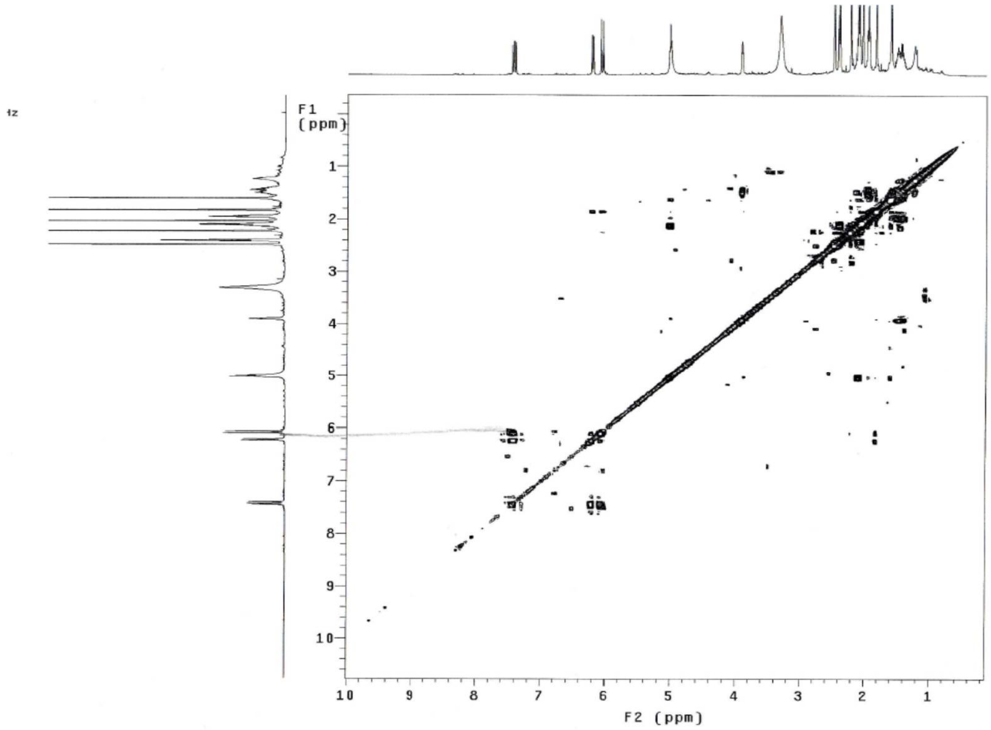

Figure S23. ^{1}H-^{1}H COSY Spectrum of Compound **2** (DMSO-d_6, 500 MHz)

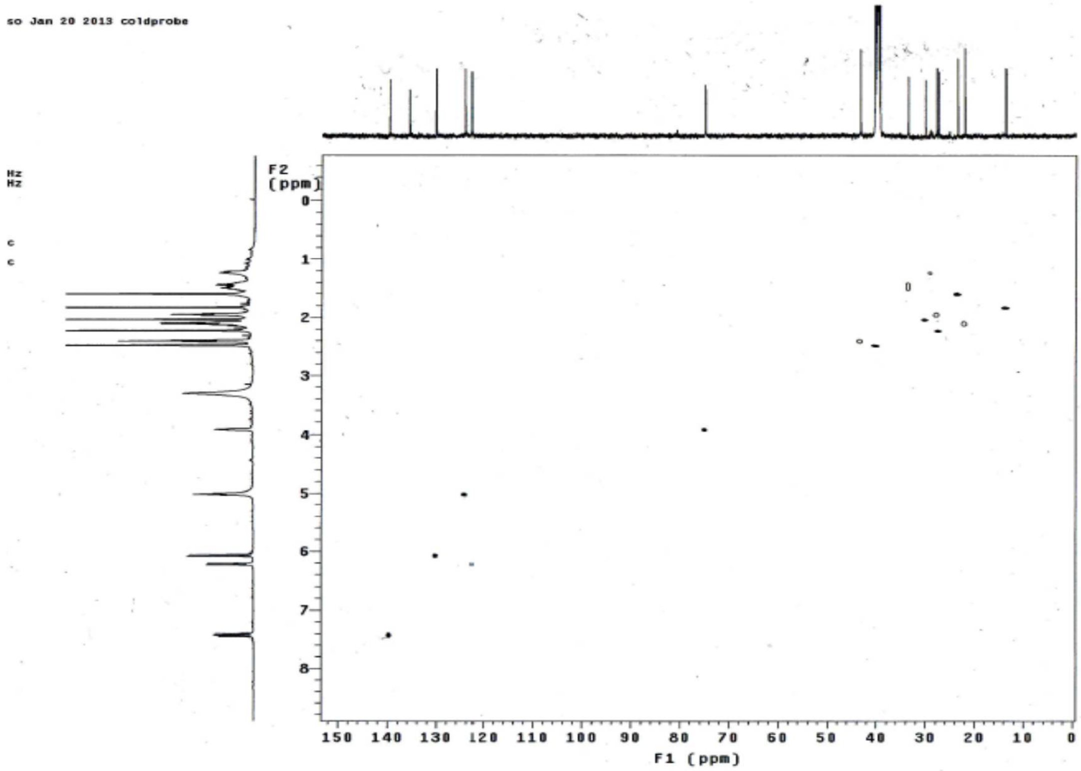

Figure S24. HSQC Spectrum of Compound **2** (500 MHz, DMSO-d_6)

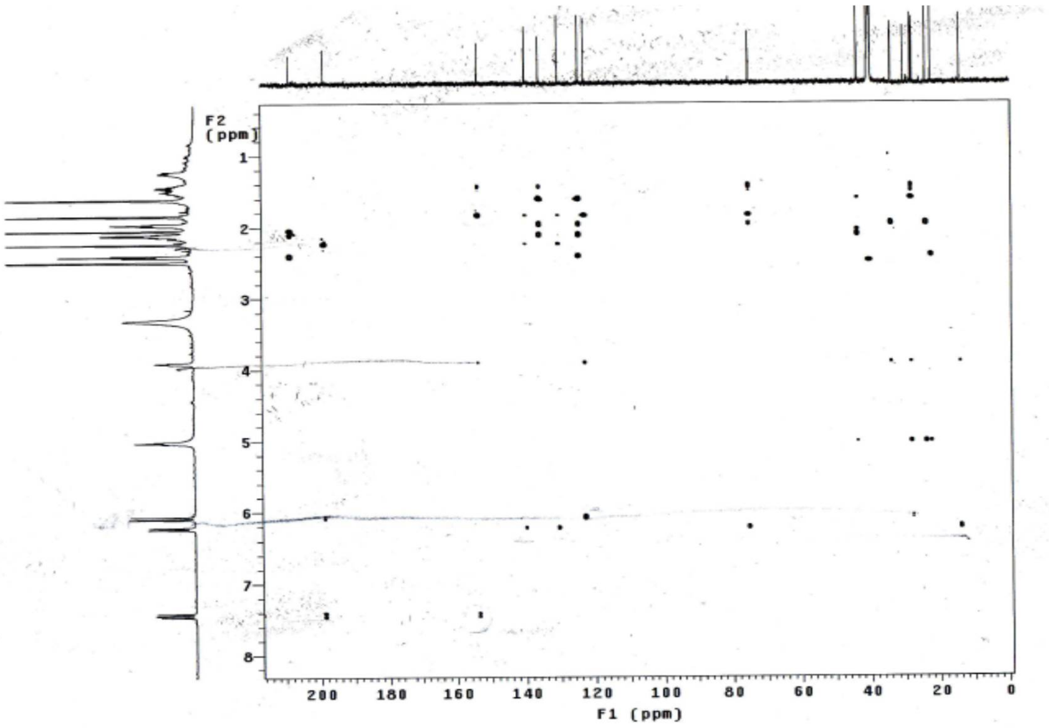

Figure S25. HMBC Spectrum of Compound **2** (500 MHz, DMSO-d_6)

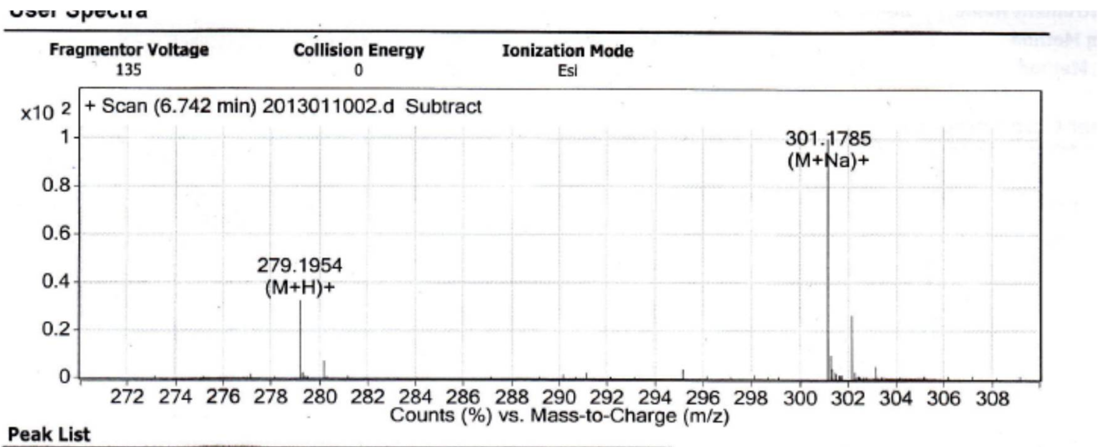

Peak List

m/z	z	Abund	Formula	Ion
185.1171		1085072		
243.1643		870144		
261.1847	1	1972335		
265.1411	1	2443816		
279.1954	1	2286441	C17 H27 O3	(M+H)+
301.1785	1	7067041	C17 H26 Na O3	(M+Na)+

Figure S26. HR-ESIMS Spectrum of Compound **2**

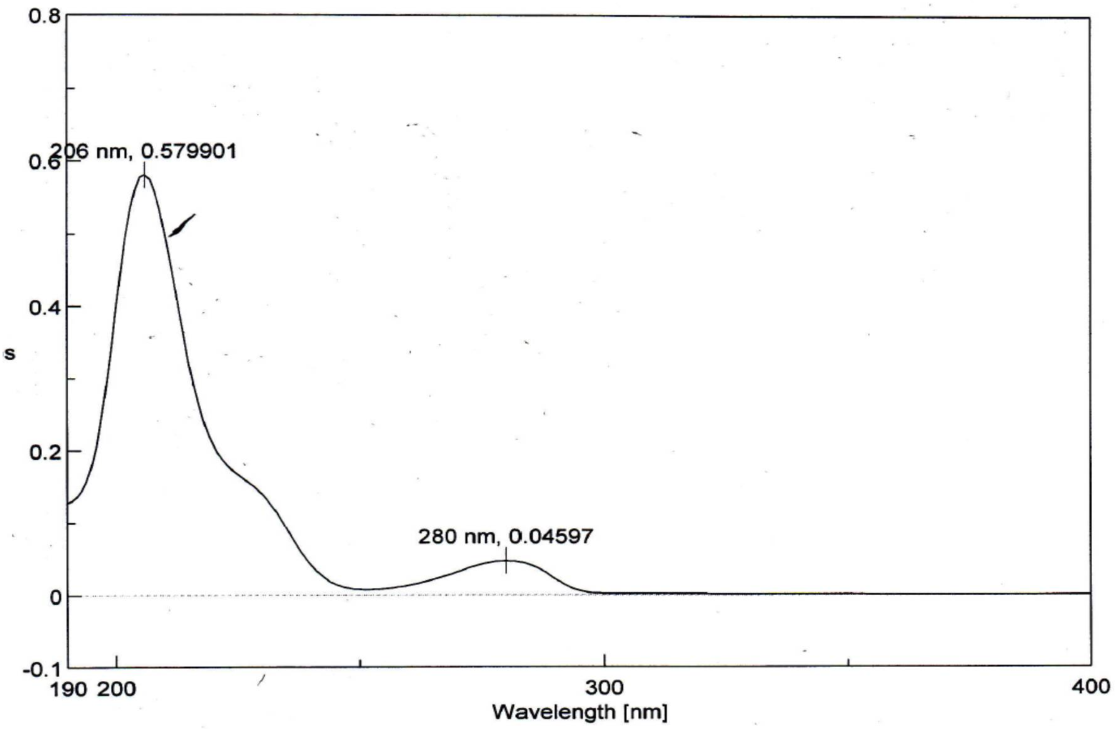

Figure S27. UV Spectrum of Compound **3**

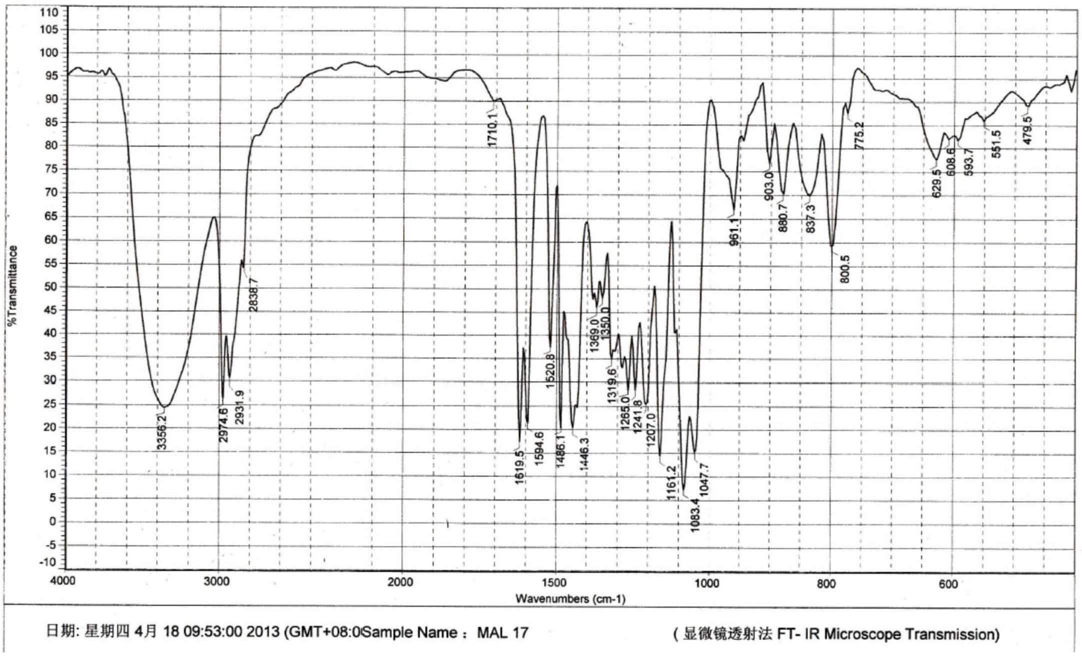

Figure S28. IR Spectrum of Compound **3.**

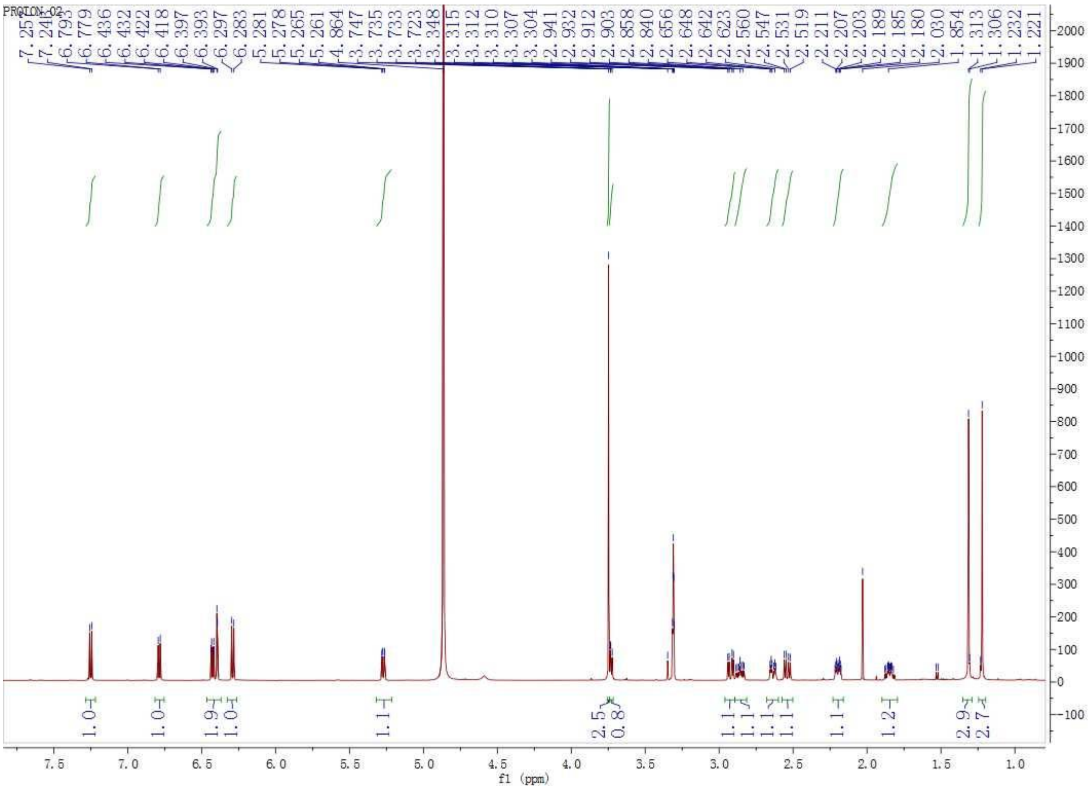

Figure S29. ^{1}H-NMR Spectrum of Compound **3** (600 MHz, CD_3OD).

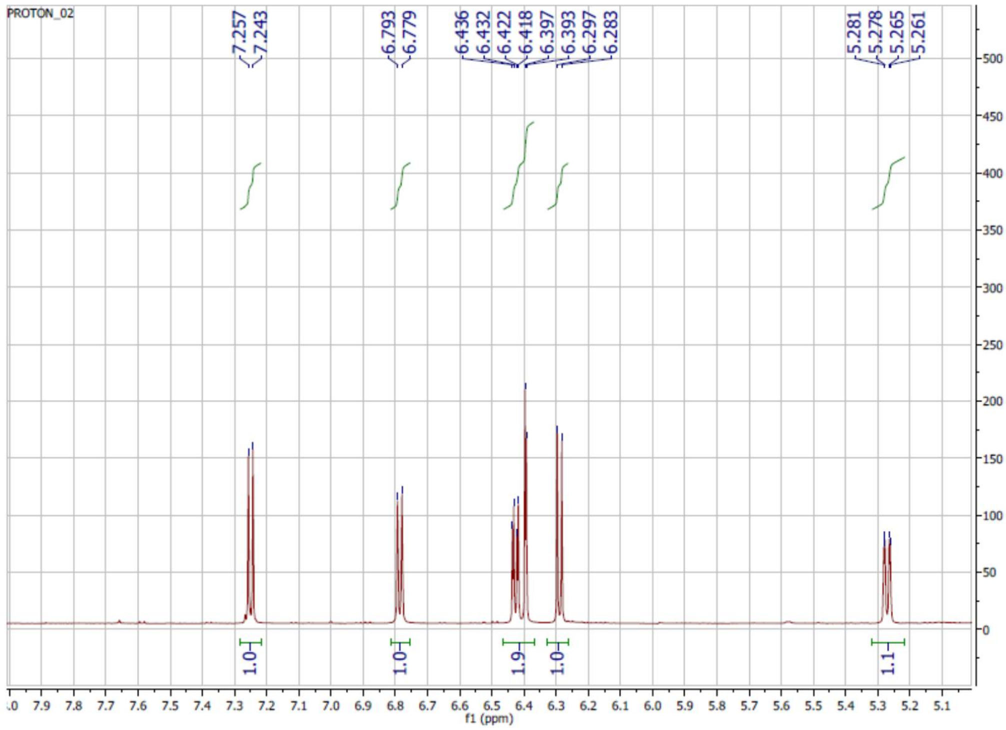

Figure S30. Enlarged ^{1}H-NMR Spectrum of Compound **3** (600 MHz, CD_3OD).

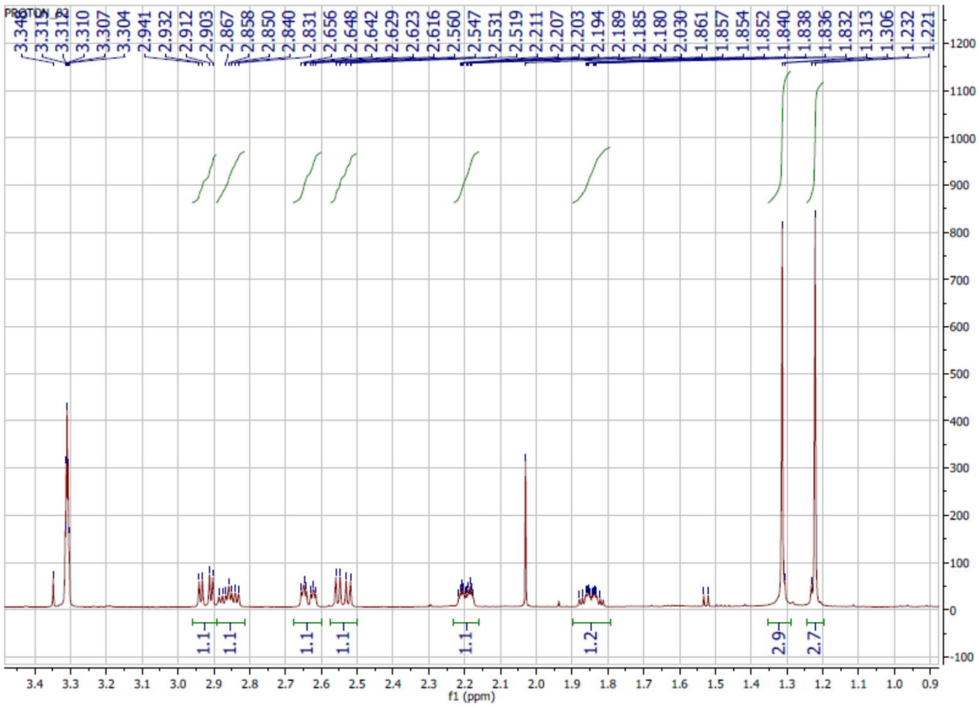

Figure S31. Enlarged ^{1}H-NMR Spectrum of Compound **3** (600 MHz, CD_3OD).

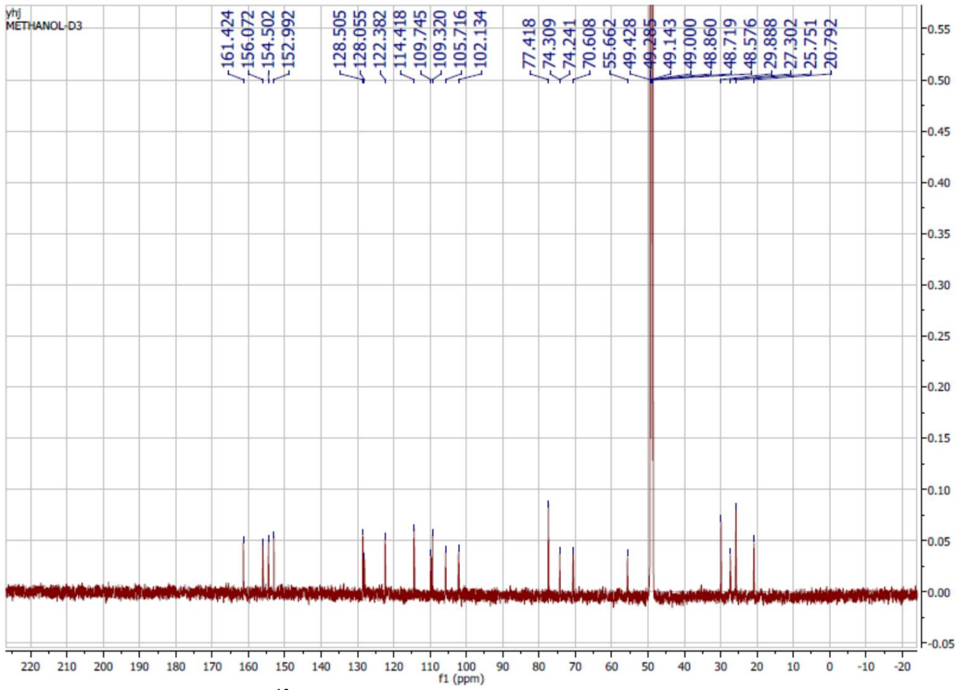

Figure S32. ^{13}C-NMR Spectrum of Compound **3** (150 MHz, CD_3OD).

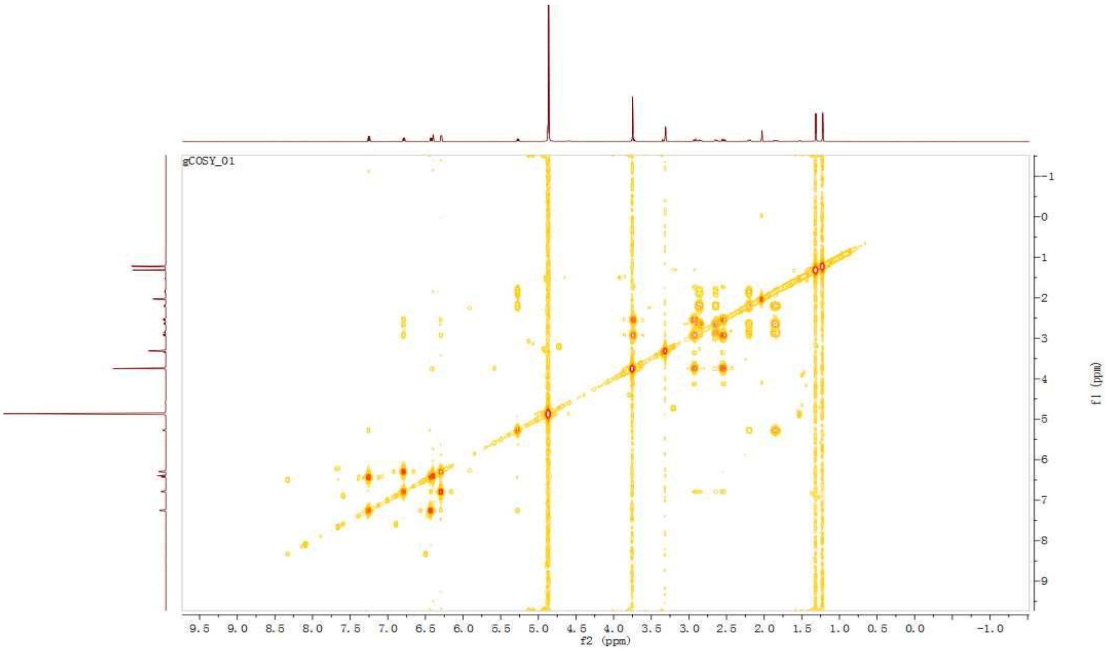

Figure S33. ^1H-^1H COSY Spectrum of Compound **3** (CD_3OD, 600 MHz).

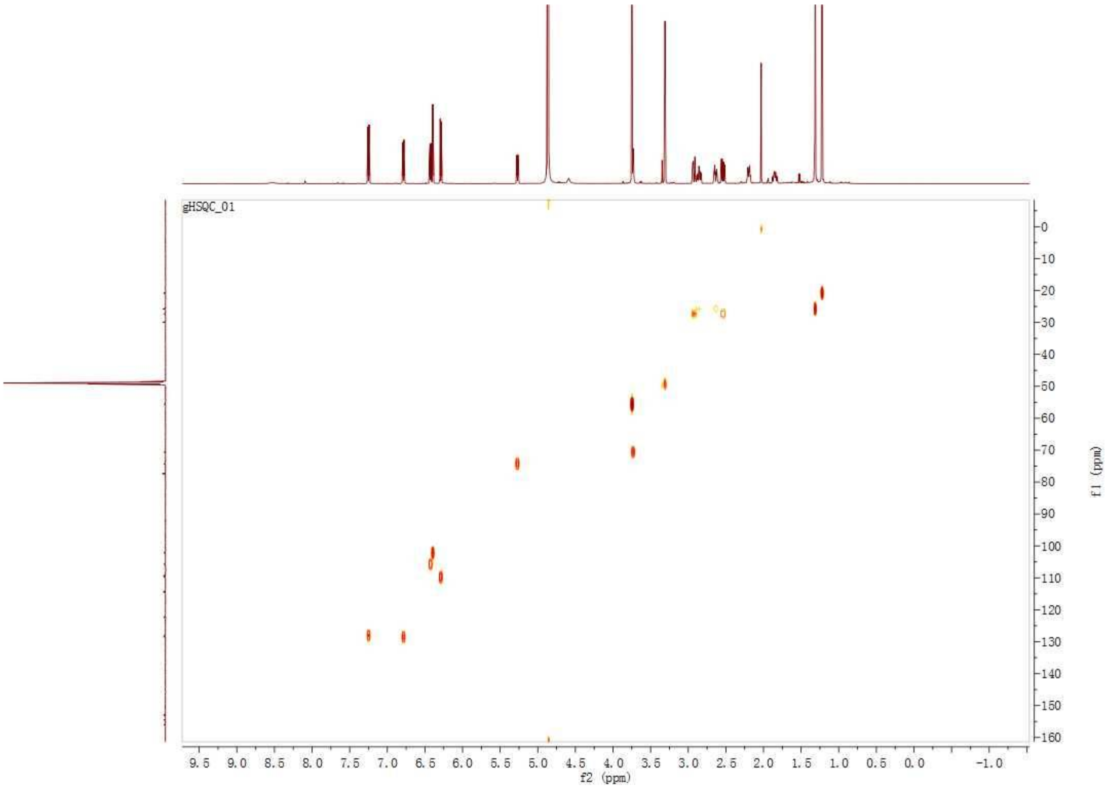

Figure S34. HSQC Spectrum of Compound **3** (600 MHz, CD_3OD).

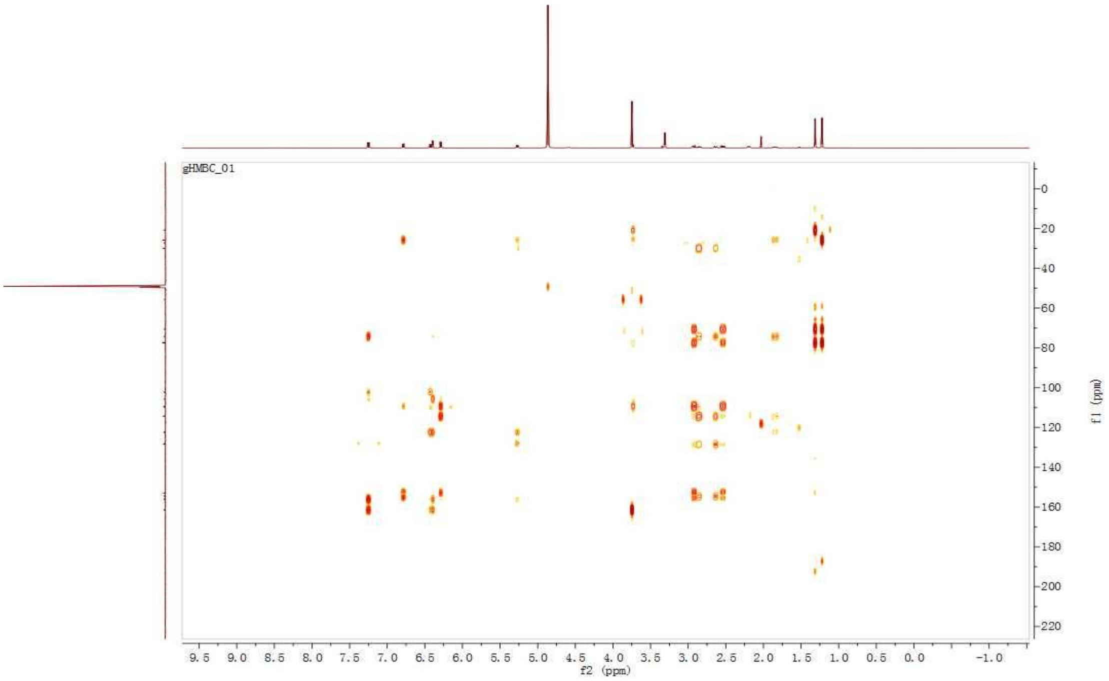

Figure S35. HMBC Spectrum of Compound **3** (600 MHz, CD_3OD).

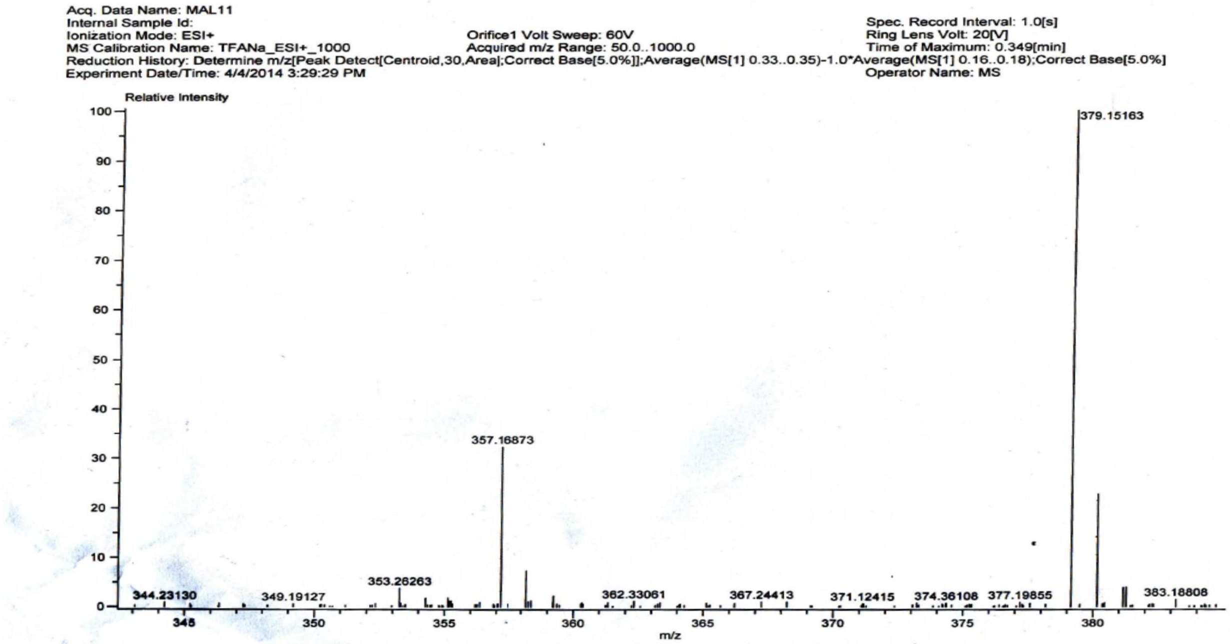

Figure S36. HR-ESIMS Spectrum of Compound **3**

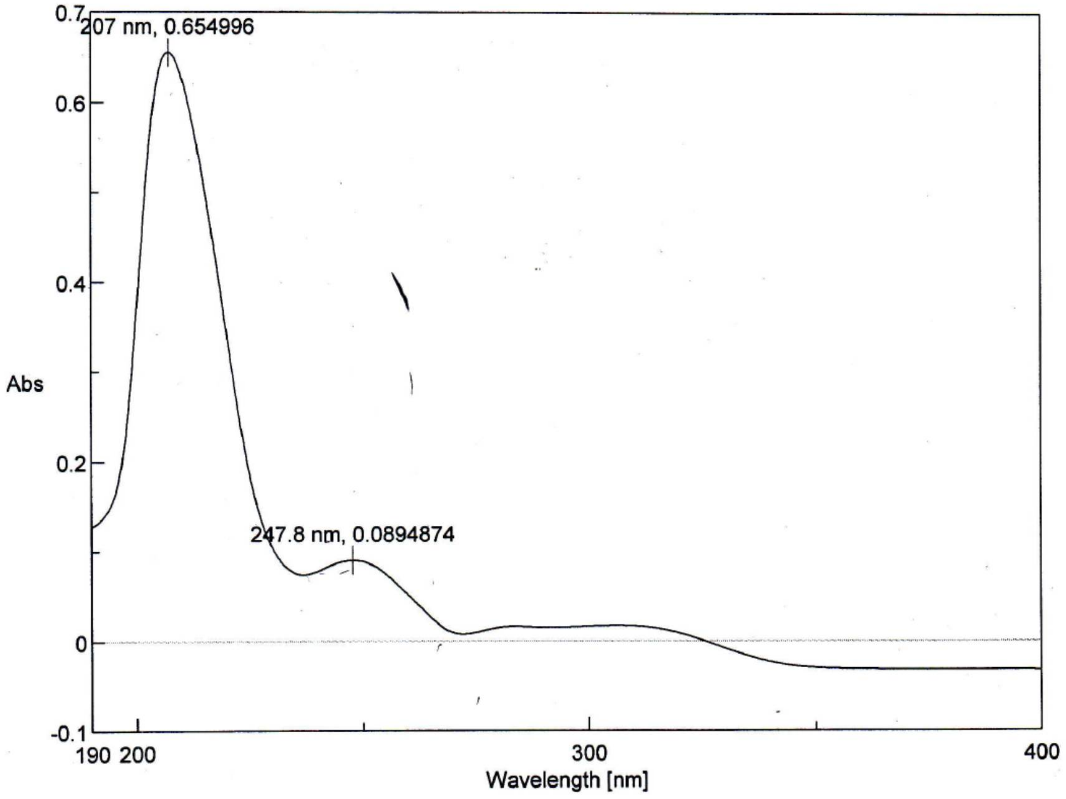

Figure S37. UV Spectrum of Compound **4.**

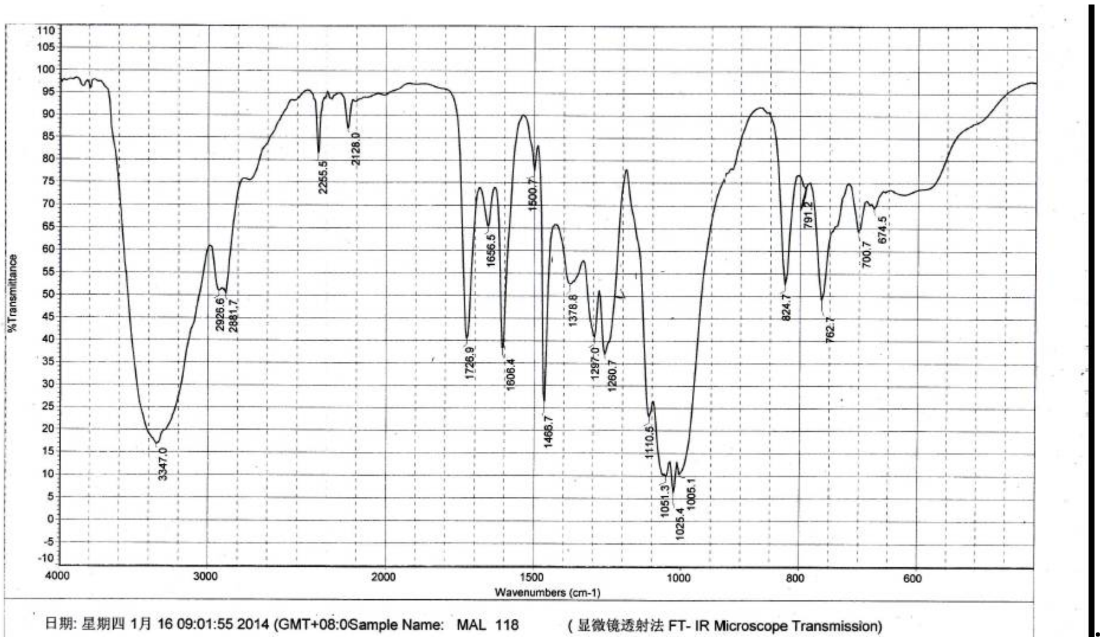

Figure S38. IR Spectrum of Compound **4.**

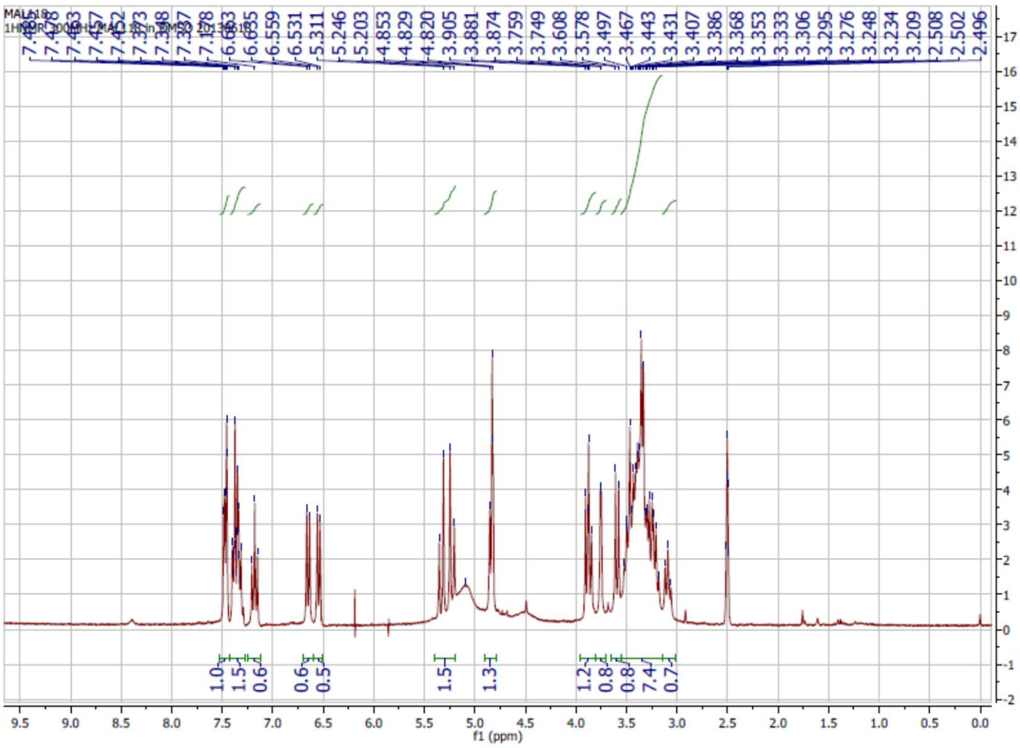

Figure S39. ^{1}H-NMR Spectrum of Compound **4** (300 MHz, DMSO-d_6).

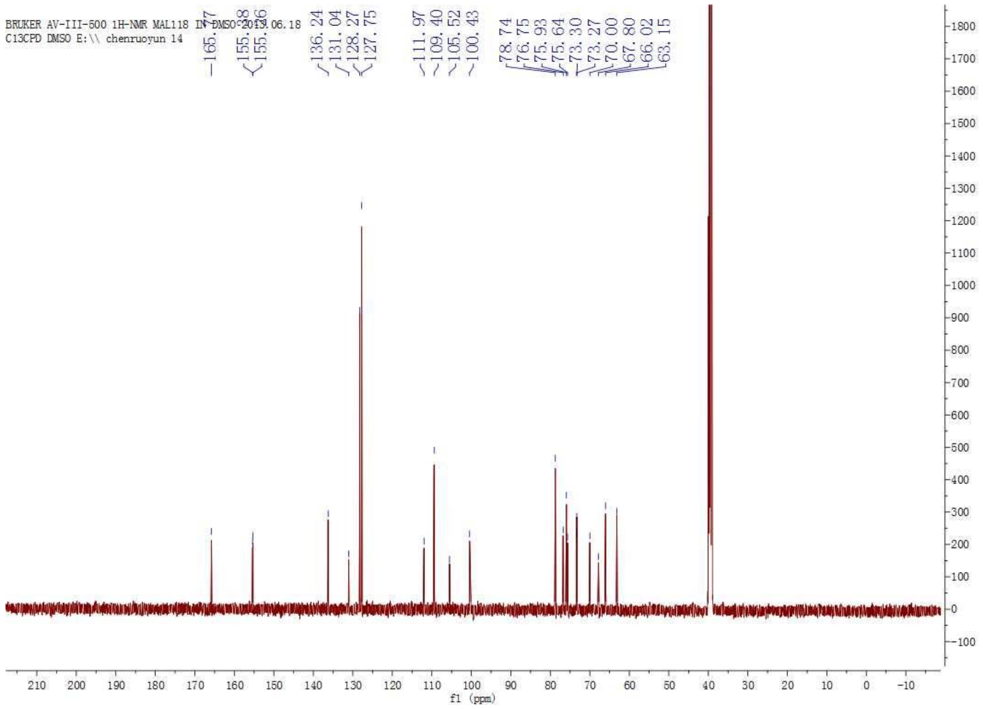

Figure S40. ^{13}C-NMR Spectrum of Compound **4** (125 MHz, DMSO-d_6).

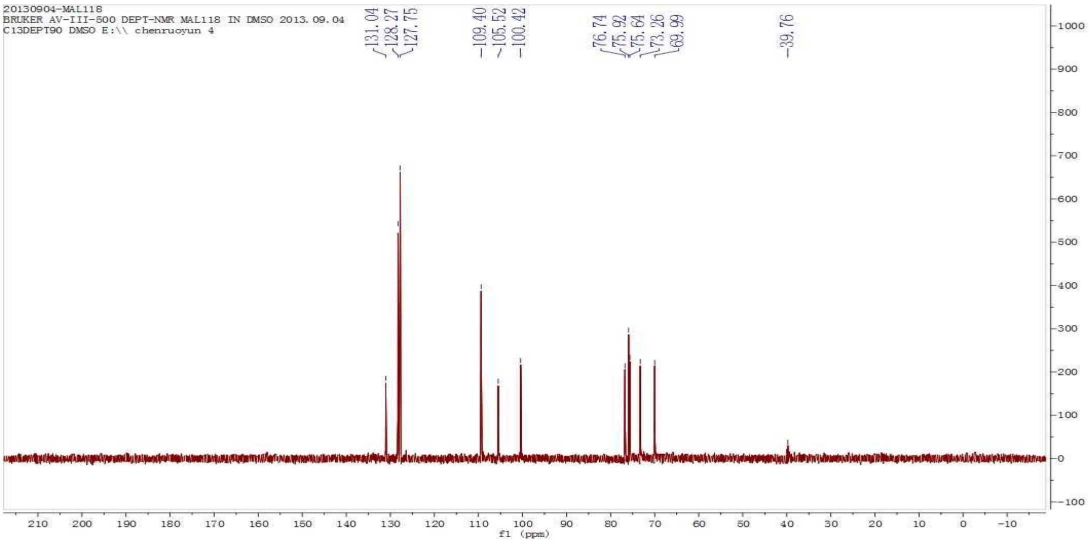

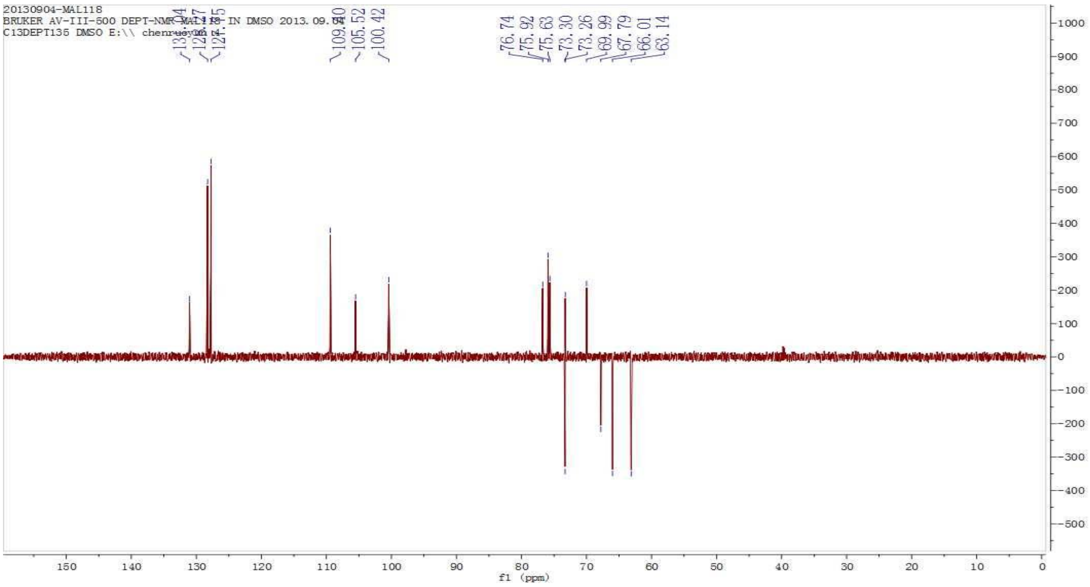

Figure S41. DEPT Spectrum of Compound **4** (125 MHz, DMSO-d_6).

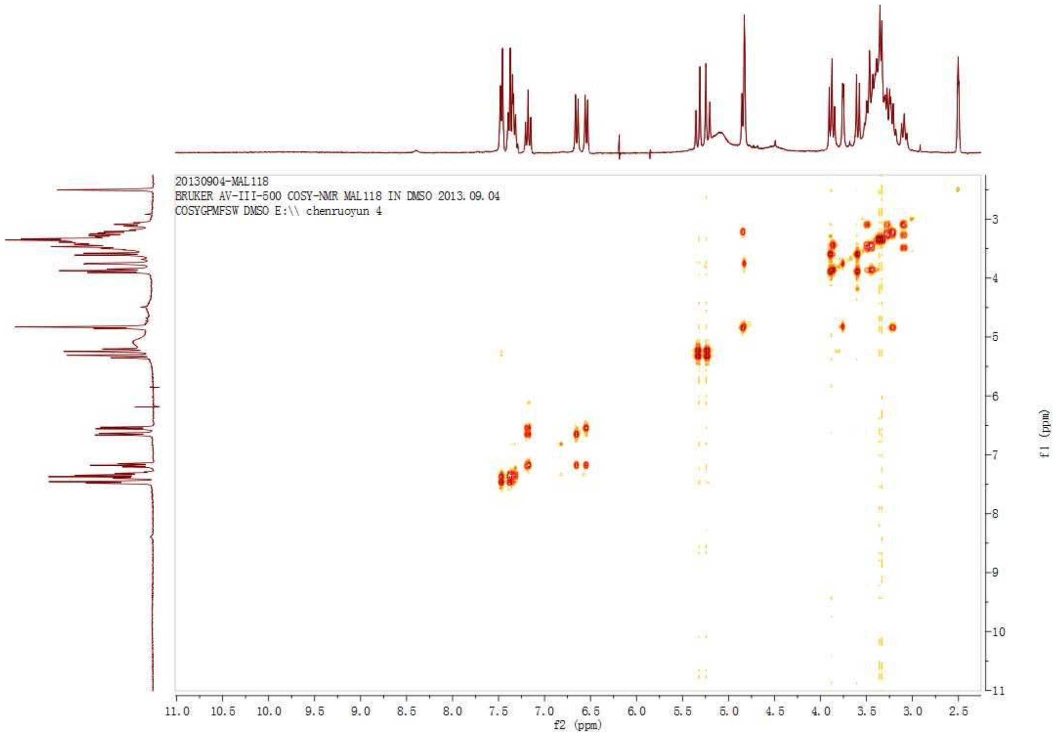

Figure S42. ^{1}H-^{1}H COSY Spectrum of Compound **4** (DMSO-*d*$_6$, 500 MHz).

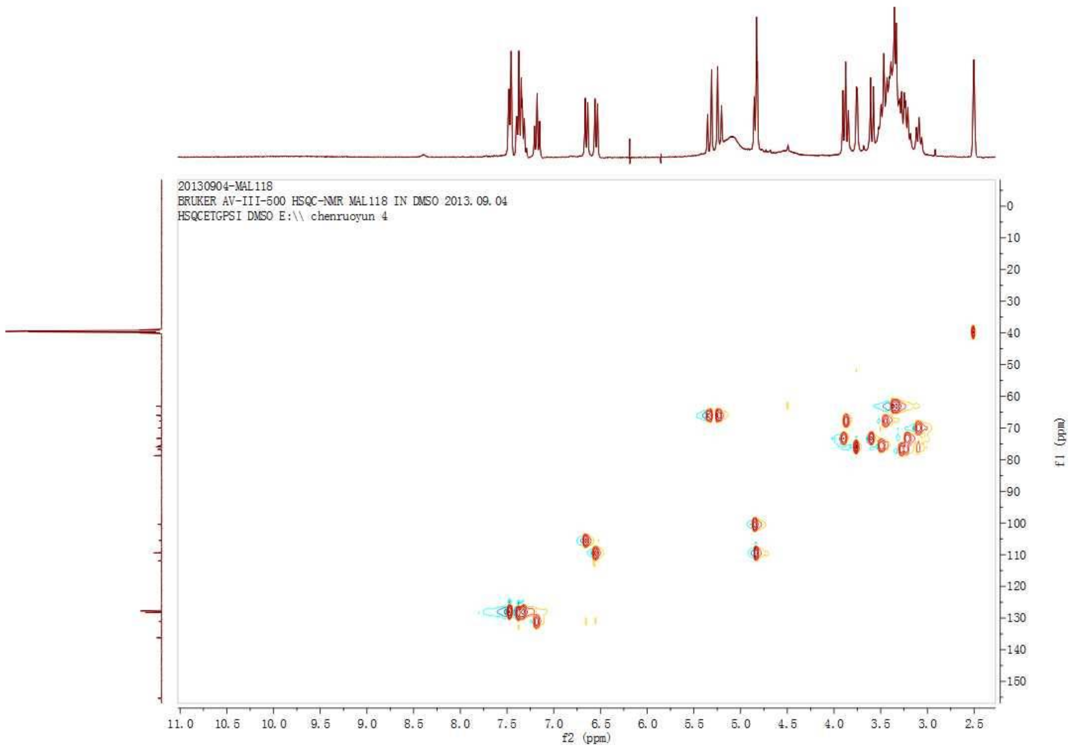

Figure S43. HSQC Spectrum of Compound **4** (500 MHz, DMSO-*d*$_6$).

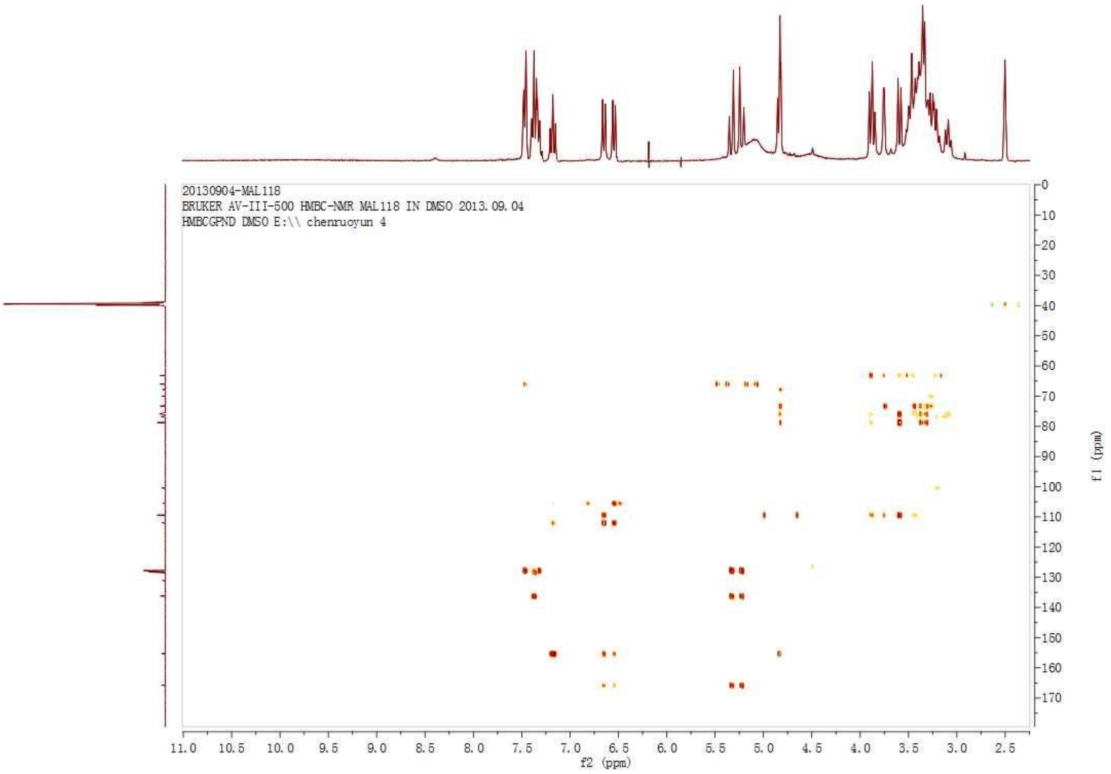

Figure S44. HMBC Spectrum of Compound **4** (500 MHz, DMSO-d_6).

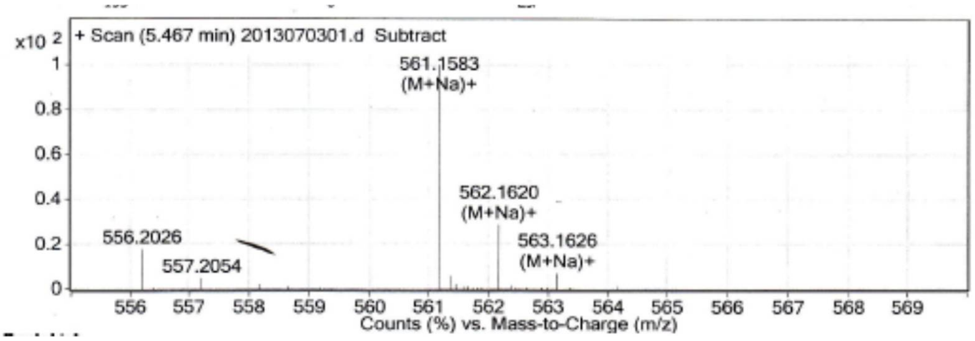

Figure S45. HR-ESIMS Spectrum of Compound **4.**

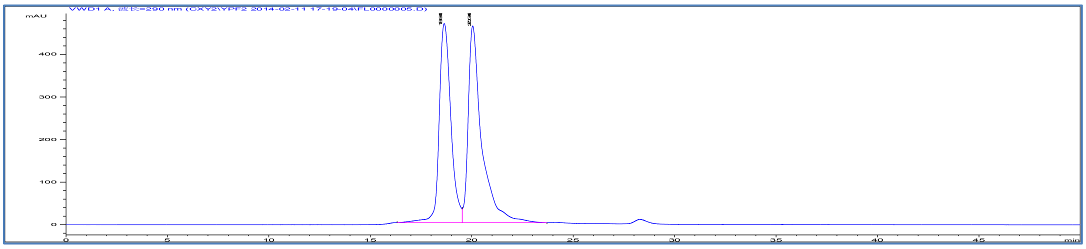

Figure S46. Compounds **2a and 2b** separated by HPLC using chiral column (Column: CHIRALPAK AD-H (150 × 4.6 mm, 5 μm); Mobile phase, hexane/isopropanol = 60:40).

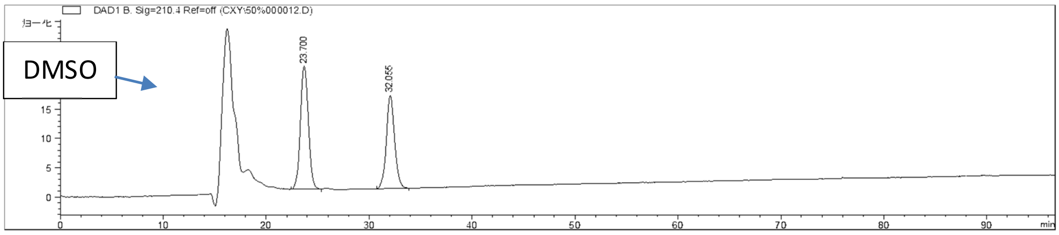

Figure S47. Compounds **3a and 3b** separated by HPLC using chiral column (Column: CHIRALPAK AD-H (150 × 4.6 mm, 5 μm); Mobile phase, hexane/isopropanol = 60:40).

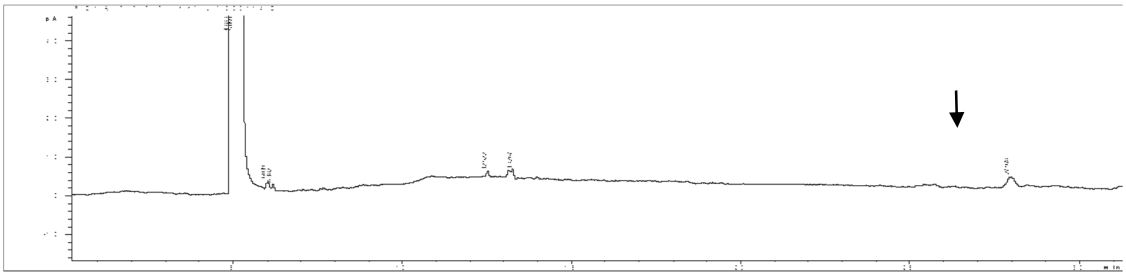

Figure S48. GC of the trimethylsilyl L-cysteine derivative of standard D-glucopyranose.

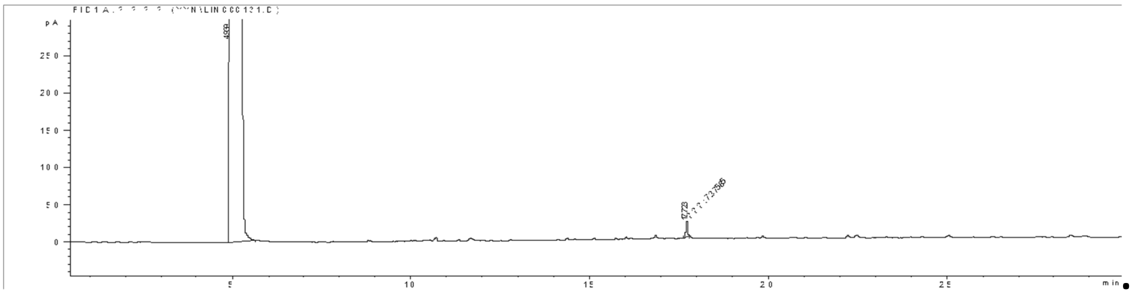

Figure S49. GC of the trimethylsilyl L-cysteine derivative of standard D-apiofuranose.

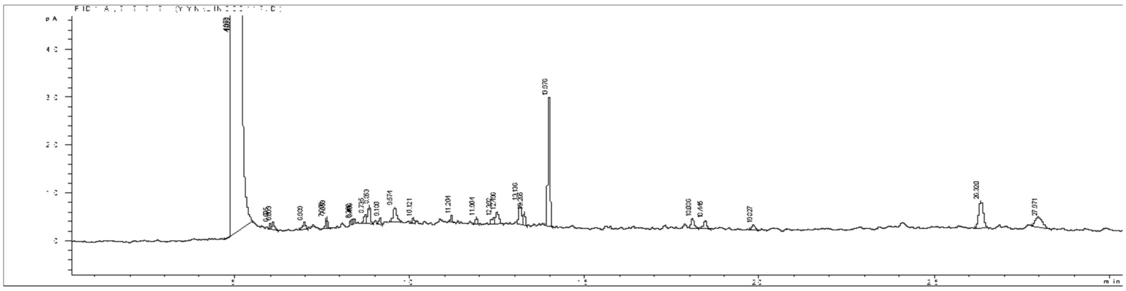

Figure S50. GC of the trimethylsilyl L-cysteine derivative of the acid hydrolysis residue of **1.**

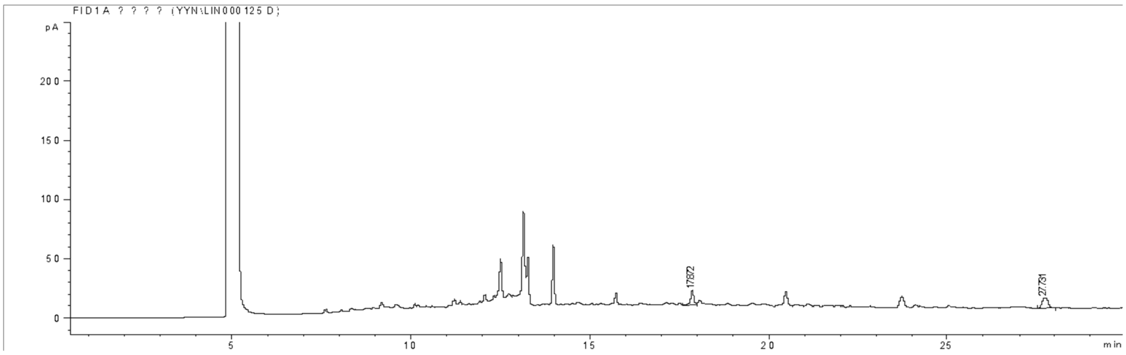

Figure S51. GC chromatography of the trimethylsilyl L-cysteine derivative of the acid hydrolysis residue of **4.**
